# Long-Term Therapy With Transcranial Magnetic Stimulation in Primary Progressive Aphasia: A Randomized Clinical Trial

**DOI:** 10.1001/jamanetworkopen.2025.26129

**Published:** 2025-08-11

**Authors:** Lucía Fernández-Romero, María Nieves Cabrera-Martin, Cristina Delgado-Alonso, Paz Suárez-Coalla, Stephanie M. Grasso, Antonio Portolés, Natalia Pérez-Macías, María Teresa Carreras, María Díez-Cirarda, María José Gil-Moreno, Javier Olazarán, Alba Vieira, Silvia Oliver-Mas, Ulises Gómez-Pinedo, Jorge Matías-Guiu, Jordi A. Matias-Guiu

**Affiliations:** 1 Department of Neurology, Hospital Clínico San Carlos, San Carlos Health Research Institute, Universidad Complutense de Madrid, Madrid, Spain; 2 Department of Nuclear Medicine, Hospital Clínico San Carlos, San Carlos Health Research Institute, Universidad Complutense de Madrid, Madrid, Spain; 3 Faculty of Psychology, University of Oviedo, Oviedo, Spain; 4 Department of Speech, Language, and Hearing Sciences, The University of Texas at Austin; 5 Department of Pharmacology and Toxicology, Universidad Complutense de Madrid, Madrid, Spain; 6 Unidad de Investigación Clínica y Ensayos Clínicos, Instituto de Investigacion Sanitaria Hospital Clinico San Carlos, Madrid, Spain; 7 Department of Neurology, Hospital La Princesa, La Princesa Health Research Institute, Madrid, Spain; 8 Department of Neurology, Hospital Gregorio Marañón, Gregorio Marañón Health Research Institute, Madrid, Spain

## Abstract

**Importance:**

Primary progressive aphasia (PPA) is a neurodegenerative clinical syndrome with no effective treatment.

**Objective:**

To evaluate the efficacy, safety, and adherence of transcranial magnetic stimulation (TMS) plus language therapy in participants with PPA over 6 months.

**Design, Setting, and Participants:**

This prospective, double-blind parallel-design randomized clinical trial was performed in Spain between December 2021 and July 2024. Participants meeting criteria for PPA and with a Clinical Dementia Rating of 0 to 1 (in which scores range from 0 to 3, with higher scores indicating greater severity of dementia symptoms) were eligible.

**Interventions:**

Participants were randomized 2:1 to active-TMS (intermittent theta-burst TMS protocol) vs sham-TMS therapy for 6 months, immediately followed by evidence-based language intervention for treating PPA, stratified by PPA variants (nonfluent/agrammatic, semantic, and logopenic).

**Main Outcomes and Measures:**

The main outcome was the standardized uptake value ratio in a large brain region encompassing most of the left hemisphere, assessed at baseline and at 6 months (immediately following the intervention) and measured by ^18^F-fludeoxyglucose–positron emission tomography. Secondary outcomes included results from the Mini Linguistic State Examination, confrontation naming, words per minute derived from a spontaneous speech task, the Interview for Deterioration in Daily Living Activities in Dementia, and the Neuropsychiatric Inventory. Secondary outcomes were assessed at baseline, 3 months, and 6 months. Safety and adherence were also evaluated.

**Results:**

Among 78 participants who were screened for the study, 63 were enrolled (mean [SD] age, 71.8 [8.4] years; 42 females [66.7%]); of these, 42 were randomized to the active-TMS group and 21 were randomized to the sham-TMS group. The primary outcome measure was positive; the adjusted mean of the standardized uptake value ratio at 6 months was higher in the active group than in the sham group (0.78 [95% CI, 0.77-0.79] vs 0.77 [95% CI, 0.75-0.78]; *P* = .046). Active TMS was associated with improvement in the mean adjusted difference for all of the secondary outcomes at 6 months (Mini Linguistic State Examination: 7.71 [95% CI, 3.06-12.35], confrontation naming: 23.81 [95% CI, 11.85-35.77], Interview for Deterioration in Daily Living Activities in Dementia: −5.39 [95% CI, −10.73 to −0.05], and Neuropsychiatric Inventory: −4.25 [95% CI, −8.37 to −0.14]), except for words per minute results (3.41 [95% CI, −8.86 to 15.68]). There were no significant differences in the number of adverse events. Adherence was complete in 58 of 63 participants (92.1%).

**Conclusions and Relevance:**

In this randomized clinical trial including participants with PPA, intermittent theta-burst TMS over the left dorsolateral prefrontal cortex during a 6-month interval was associated with less decline in regional brain metabolism and improvement in language abilities, functional independence, and neuropsychiatric symptoms. These findings suggest that TMS combined with evidence-based language intervention in participants with PPA promotes greater functional outcomes and slower progression compared with language therapy alone.

**Trial Registration:**

ClinicalTrials.gov Identifier: NCT05842473

## Key Points

**Question:**

What is the efficacy of long-term transcranial magnetic stimulation (TMS) combined with language therapy in individuals with primary progressive aphasia (PPA)?

**Findings:**

In this randomized clinical trial, which included 63 participants diagnosed with PPA, TMS administered over a 6-month period mitigated decline in regional brain metabolism, language, and functionality.

**Meaning:**

These findings suggest that long-term TMS combined with language therapy may help slow the progression of PPA and offer a new approach for the treatment of language disturbances due to neurodegenerative disorders.

## Teaser

This randomized clinical trial assesses whether long-term transcranial magnetic stimulation (TMS) combined with language therapy is effective in treating primary progressive aphasia progression.

## Introduction

Primary progressive aphasia (PPA) is a neurodegenerative clinical syndrome with insidious onset characterized by prominent speech and/or language impairment.^1^ It is a heterogeneous syndrome that can be the mode of presentation of Alzheimer disease (AD) and frontotemporal degeneration,^2^ which are 2 of the most common causes of dementia. According to the current international consensus criteria,^3^ 3 variants of PPA are recognized: nonfluent/agrammatic (nfvPPA), semantic (svPPA), and logopenic (lvPPA). The first 2 are generally included in the group of frontotemporal degeneration, while lvPPA is associated with AD. This syndrome impacts individuals’ communication and quality of life, and currently, there are no effective pharmacologic treatments available, although nonpharmacologic speech-language intervention has proven to be beneficial.^4^

In recent years, noninvasive brain stimulation techniques, such as transcranial magnetic stimulation (TMS) or transcranial direct current stimulation, have been explored as a potential treatment for neurodegenerative diseases^5^ and, in particular, for PPA.^6,7^ TMS modulates cortical excitability and induces synaptic and nonsynaptic changes.^8^ These changes can lead to modifications in cognition and neurotransmitters, as well as in brain neuroplasticity.^9^ TMS is used as a treatment for other diseases such as depression and poststroke aphasia.^10,11,12^ Previous studies have examined the short-term effects of TMS on PPA, reporting some improvements in naming, some cognitive tests, apathy, spontaneous speech, and regional cerebral metabolism.^13,14,15,16^ These results are encouraging and suggest that TMS may be a promising treatment, but, to our knowledge, the longer-term effects (eg, more than a few weeks of intervention) have not been examined.

This study aimed to compare the efficacy of TMS plus language therapy with sham TMS plus an evidence-based form of language intervention in the progression of PPA, as determined by the changes in brain metabolism measured by ^18^F-fludeoxyglucose–positron emission tomography (FDG-PET) at 6 months. In addition, we aimed to examine the effect of TMS on language abilities, functional independence, and neuropsychiatric symptoms.

## Methods

### Participants and Study Design

We conducted a prospective, double-blind, parallel-design randomized clinical trial (Long-Term Effect of TMS in Primary Progressive Aphasia [RECONNECT]) to evaluate the efficacy of active TMS combined with language therapy compared with sham TMS plus language therapy on brain metabolism, language, functional abilities, and neuropsychiatric symptoms in participants with PPA. The study was conducted at the Department of Neurology of the Hospital Clínico San Carlos in Madrid, Spain, from December 2021 to July 2024. The trial protocol is included in Supplement 1 and was approved by the hospital ethics committee and the Spanish Agency of Medicines and Medical Devices. All participants provided written informed consent for study inclusion. We followed the Consolidated Standards of Reporting Trials (CONSORT) reporting guideline.

**Figure 1.**
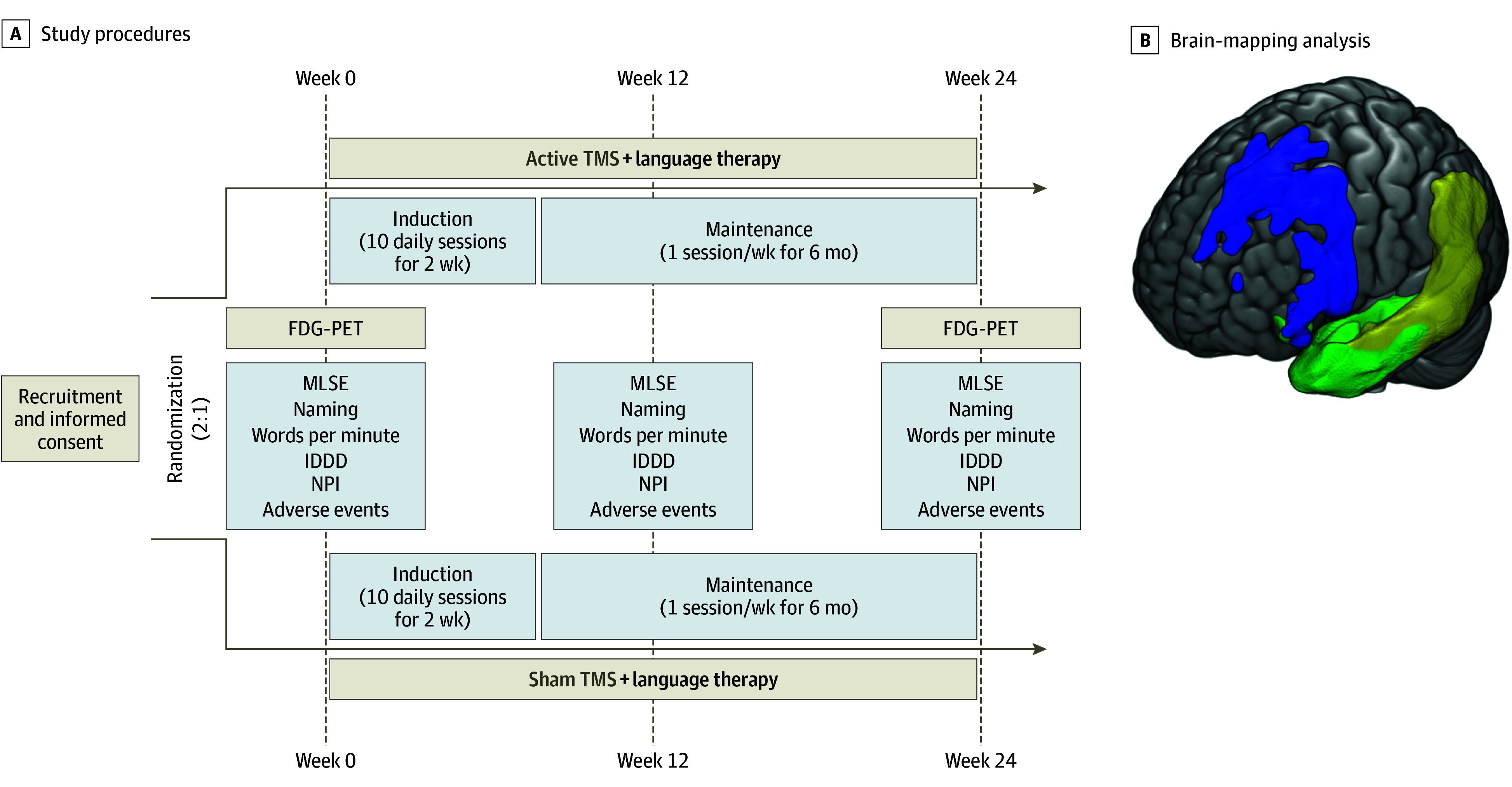
Summary of the Study Design A, The trial consisted of a 24-week treatment that included a 2-week intensive course, in which active transcranial magnetic stimulation (TMS) or sham TMS combined with language therapy was applied daily, followed by a maintenance phase in which the same stimulation was applied weekly for 22 weeks. Participants were assessed at baseline, 3 months after the start of treatment, and at the end of the treatment (6 months following baseline). B, A brain image displays the results of a voxel-based brain-mapping analysis of participants included in the trial, classified by the primary progressive aphasia (PPA) variant, compared with a healthy control group of 24 participants. Regions of reduced metabolism are shown (blue, nonfluent or agrammatic variant PPA; green, semantic variant PPA; and yellow, logopenic variant PPA) with cluster-level family-wise error rate correction applied. FDG-PET indicates ^18^F-fludeoxyglucose–positron emission tomography; IDDD, Interview for Deterioration in Daily Living Activities in Dementia; MLSE, Mini Linguistic State Examination; NPI, Neuropsychiatric Inventory.

Participants were eligible if they had an established diagnosis of PPA according to criteria in the study by Gorno-Tempini et al,^3^ had a Clinical Dementia Rating score of 0 to 1 (scores range from 0 to 3, with higher scores indicating greater severity of dementia symptoms), language was the participant’s primary deficit, and informed consent was provided by the participant or their legal guardian. Race and ethnicity data were collected via self-report and were included in the study to document participant demographics. Exclusion criteria were the following: a diagnosis of a disease other than PPA with potential impact on language, history of epilepsy, or implanted cranial or thoracic devices or any other contraindication to TMS or magnetic resonance imaging; breastfeeding, pregnant, or planning pregnancy within the next year; a terminal medical condition with a life expectancy of less than 1 year; any malignant disease within the last 2 years; alcohol or substance use disorder in the past year; psychiatric disorders; absolute inability to communicate (mutism) or poor language proficiency that would hinder study participation; severity of PPA that would prevent following the study interventions or assessments at the time of inclusion; participation in another clinical trial within the previous 4 months; or chronic use of medications that may have influenced the study outcomes. The procedures of the study are summarized in Figure 1A, with a more detailed description provided in the eAppendix in Supplement 2.

### Randomization and Blinding

Participants were randomly allocated at a 2:1 ratio into 2 groups (active TMS or sham TMS) using a web-based random number generator.^17^ The randomization was stratified by clinical variant (nvPPA, svPPA, or lvPPA). Both participants and the assessor (C.D.-A.) were blinded to the allocation. The researcher performing the TMS sessions did not have access to the assessment results.

### Assessments and Outcomes

For the primary outcome measures, participants were assessed at baseline and at 6 months after starting treatment, which occurred immediately after the last stimulation session. The primary outcome measure was the standardized uptake value ratio (SUVR) at 6 months, which described the change in the regional brain metabolism between the baseline and final visit, assessed by FDG-PET. The secondary outcome measures, assessed at baseline; 3 months after starting treatment, coinciding with the midpoint of the treatment; and at 6 months after starting treatment, included results from (1) the Mini Linguistic State Examination (MLSE), (2) confrontation naming of a list of trained objects, (3) words per minute derived from a spontaneous speech task, (4) functional activity assessed using the Interview for Deterioration in Daily Living Activities in Dementia (IDDD) scale, and (5) neuropsychiatric symptoms assessed using the Neuropsychiatric Inventory (NPI).

### TMS

The stimulation protocol included a 6-month treatment period, beginning with an intensive period of 10 sessions over 2 consecutive weeks with a break over the weekend between weeks, followed by a 22-week maintenance phase, in which the same treatment was applied once weekly. All sessions used an intermittent theta-burst protocol: a total of 600 pulses at 50 Hz, distributed across 20 cycles of 3 pulses with an interval of 10 seconds. This protocol lasted for approximately 3 minutes. Treatment was delivered at 120% of the resting motor threshold, with a maximum of 50% of the maximum stimulator output. Brain stimulation was applied under neuronavigation using a Rapid^2^ stimulator (Magstim) with a figure-8 coil over the left dorsolateral prefrontal cortex (DLPFC). For sham TMS, a sham coil was used, which is indistinguishable from the active-TMS coil. The sessions were carried out with the same frequency and the same duration on the same target with neuronavigation.

### Language Treatment and Stimuli Selection

We used an adapted form of lexical retrieval treatment (LRT)^18,19,20,21^ for all enrolled participants, regardless of treatment condition (active or sham). LRT is based on the training of lexical retrieval strategies that engage and strengthen residual semantic, orthographic, and phonologic knowledge and has previously been proven effective in the treatment of PPA in the absence of neuromodulation.^18,20^ The approach consists of a sequence of tasks to guide the engagement, strengthening, and active use of central components of language processing, beginning with training semantic self-cueing techniques and progressing through orthographic and phonemic self-cues. The specific sequence of training tasks is described in further detail in eTable 1 in Supplement 2.

All therapy sessions were delivered in person by a neuropsychologist (L.F.-R. and S.O.-M.) with clinical experience (5 years) in the assessment and treatment of individuals with PPA, who received specific training in the LRT protocol. During the first 2 weeks, the participant received 10 language treatment sessions. Subsequently, each participant received 1 session per week over the 6-month treatment period. The language treatment sessions were administered immediately after the TMS session. The intervention sessions lasted approximately 50 minutes.

One set of 5 nouns was trained per session. The words to be trained were selected from a list of 261 words from 8 different semantic categories: animals, foods, objects, places, furniture, clothing, transports, and body parts. Two sessions of oral naming were conducted before treatment began. Only nouns that were not named correctly or were unnamed on both occasions were eligible for training. Each treatment day began with a new set of 5 nouns. When the entire eligible training list ran out of new nouns, we cycled through the eligible sets again.

After the initial 2-week period, daily homework was introduced for each set. Participants completed the homework independently at home using slide materials that were sent via email weekly. These slides included a picture of the trained targets from the session and its associated written form. The participants, or caregivers when support was needed, were instructed to copy the written word and produce the spoken word aloud 10 times, following Copy and Recall Treatment,^22^ the home-based practice often paired with previous reports of LRT.

### Sample Size Calculation

The sample size calculation was based on the primary end point. In a sample of 70 participants with PPA who were longitudinally evaluated with 2 FDG-PET studies, we estimated a linear regression for the rate of decline in brain metabolism in a region of interest covering a large part of the left hemisphere. Assuming that the treatment could reduce this decline in brain metabolism by at least 20%, the estimated required sample size was 54 participants. Considering a 10% dropout rate, the final sample size was established as 60 participants.

### Statistical Analysis

The original protocols of the study were stored in a locked office. Deidentified data were saved on a secure electronic database. We used REDCap for data collection.

Statistical analyses were performed using SPSS Statistics, version 26.0 (IBM Inc). Descriptive data are shown as mean (SD), absolute frequency (percentage), and 95% CI. Normality was checked using the Shapiro-Wilk test. Baseline and demographic characteristics were presented.

To assess the effect of the intervention, an analysis of covariance (ANCOVA) was performed. The ANCOVA included a dependent variable of each primary and secondary end point (eg, SUVR at 6 months or MLSE at 6 months), and the primary independent variable was the treatment group (active vs sham TMS). PPA variant and sex were also included as factors. Baseline measurements of the dependent variable and SUVR at baseline for the secondary outcomes were included as covariates. The model assumptions, including normality of residuals and homogeneity of variances, were checked. Effect sizes were evaluated with partial η^2^. Additional ANCOVA models were also estimated for the secondary outcomes to evaluate the mean adjusted difference at 3 months, also controlling for PPA variant, sex, baseline SUVR, and baseline assessment. Mann-Whitney tests were used to compare continuous, nonnormally distributed variables between groups, and χ^2^ tests were used to analyze categorical variables.

A 2-sided *P* value <.05 was used as the statistical threshold. The final analysis was conducted according to the intention-to-treat principle, including all of the randomized participants. However, because we did not have data for 3 participants due to early withdrawal, the missing data were not imputed, and the analysis was conducted with the data available (modified intention-to-treat analysis).

## Results

### Participant Characteristics

**Figure 2.**
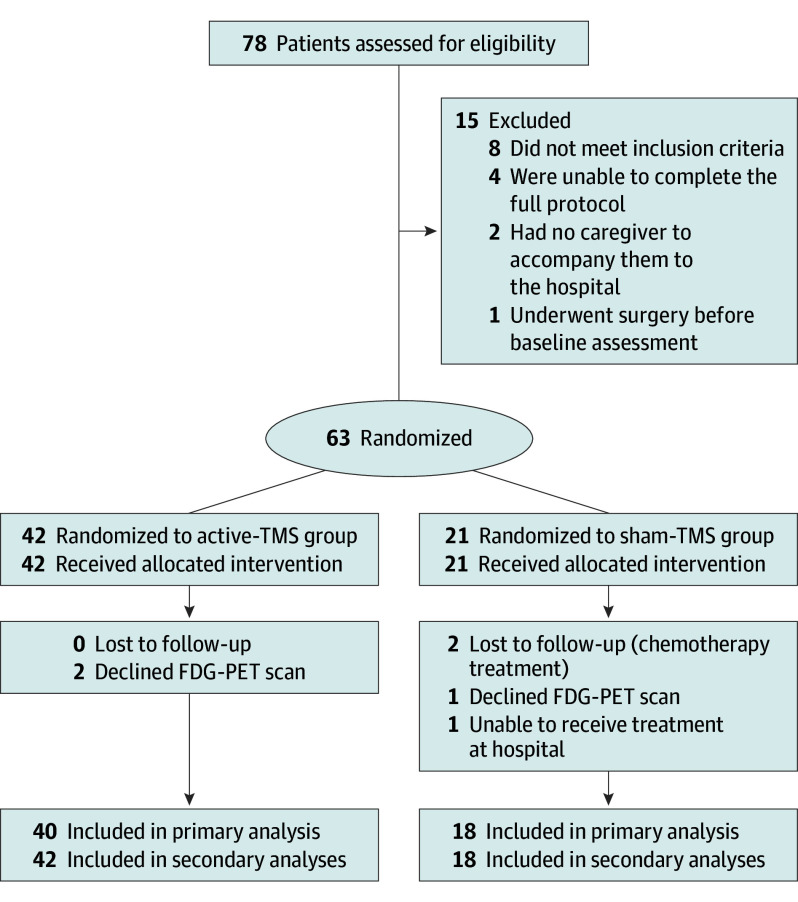
Participant Flow Diagram FDG-PET indicates ^18^F-fludeoxyglucose–positron emission tomography; TMS, transcranial magnetic stimulation.

Baseline characteristics of participants are shown in the Table and in eTables 2 and 3 in Supplement 2. Between December 2021 and June 2024, 78 participants were assessed for eligibility, and 63 participants (80.8%) were enrolled (mean [SD] age, 71.8 [8.4] years; 42 females [66.7%] and 21 males [33.3%]) and randomly assigned to each variant group (24 [38.1%] with nfvPPA, 12 [19.0%] with svPPA, and 27 [42.9%] with lvPPA) (Figure 2).

**Table.** Participant Characteristics at Baseline^a^

Characteristic	TMS Group	
	Active (n = 42)	Sham (n = 21)
**Demographic**		
Age, mean (SD), y	71.85 (8.35)	71.61 (8.59)
Sex		
Female	31 (73.8)	11 (52.4)
Male	11 (26.2)	10 (47.6)
White race	42 (100.0)	21 (100.0)
Education, mean (SD) y	13.76 (5.06)	13.57 (4.02)
First language		
Spanish	41 (97.6)	20 (95.2)
English	1 (2.4)	1 (4.8)
**Clinical**		
Time since symptom onset, mean (SD), mo	29.17 (17.88)	31.42 (21.02)
PPA variant		
nfvPPA	17 (40.5)	7 (33.3)
svPPA	8 (19.0)	4 (19.0)
lvPPA	17 (40.5)	10 (47.6)
SUVR, mean (SE)	0.83 (0.01)	0.74 (0.02)
ACE-III, mean (SD)^b^	55.12 (18.80)	42.33 (21.43)
MLSE, mean (SD)	78.93 (11.18)	73.43 (16.26)
Naming, mean (SD)	122.21 (11.63)	127.12 (15.66)
WPM, mean (SD)	68.59 (6.12)	67.47 (8.25)
NPI, mean (SD)	8.93 (11.82)	7.76 (12.45)
Depression	8 (19.0)	4 (19.0)
IDDD, mean (SD)	48.02 (14.35)	45.45 (10.45)
Speech therapy	11 (26.2)	7 (33.3)
**Comorbidities**		
Dyslipidemia	5 (11.9)	4 (19.0)
Arterial hypertension	5 (11.9)	4 (19.0)
Diabetes	3 (7.1)	2 (9.5)
Atrial fibrillation	1 (2.4)	1 (4.8)
Other cardiopathies	1 (2.4)	1 (4.8)
**Concomitant therapies**		
Cholinesterase inhibitors	5 (11.9)	4 (19.0)
Memantine	1 (2.4)	1 (4.8)
Levodopa	1 (2.4)	0
Antipsychotics	3 (7.1)	1 (4.8)
Selective serotonin reuptake inhibitors	7 (16.7)	7 (33.3)

Abbreviations: ACE-III, Addenbrooke’s Cognitive Examination-III (scores range from 9 to 100, in which higher scores indicate more preserved cognition); IDDD, Interview for Deterioration of Daily Living in Dementia; lvPPA, logopenic variant primary progressive aphasia; MLSE, Mini Linguistic State Examination; nfvPPA, nonfluent/agrammatic variant primary progressive aphasia; NPI, Neuropsychiatric Inventory; PPA, primary progressive aphasia; SUVR, standardized uptake value ratio; svPPA, semantic variant primary progressive aphasia; TMS, transcranial magnetic stimulation; WPM, words per minute.

^a^ Data are presented as No. (%) of participants unless indicated otherwise.

^b^ Total score.

Forty-two participants (66.7%) received active TMS, and 21 (33.3%) received sham TMS. Both active-TMS and sham-TMS groups were well matched, with a mean (SD) age of 71.8 (8.4) years and a mean (SD) 13.8 (5.1) years of education for the active-TMS group and a mean (SD) age of 71.6 (8.6) years and mean (SD) 13.6 (4.0) years of education for the sham-TMS group. There were 31 females (73.8%) in the active-TMS group and 11 (52.4%) in the sham-TMS group. The last individual was randomized in January 2024. In terms of race, all 63 participants (100%) were White.

From the sham-TMS group, 2 participants were lost to follow-up because they began chemotherapy treatment after 11 and 12 weeks of treatment, the primary outcome for 1 individual was lost because they elected to not undergo the FDG-PET scan, and the secondary outcomes for another participant were lost because they were unable to receive treatment at the hospital; however, the primary outcome was available. For the active-TMS group, the primary outcome for 2 individuals was lost because they elected to not undergo the FDG-PET scan. The clinical characteristics of participants who did not complete the treatment or the protocol are detailed in eTables 4 and 5 in Supplement 2.

### Efficacy

#### Primary End Point

**Figure 3.**
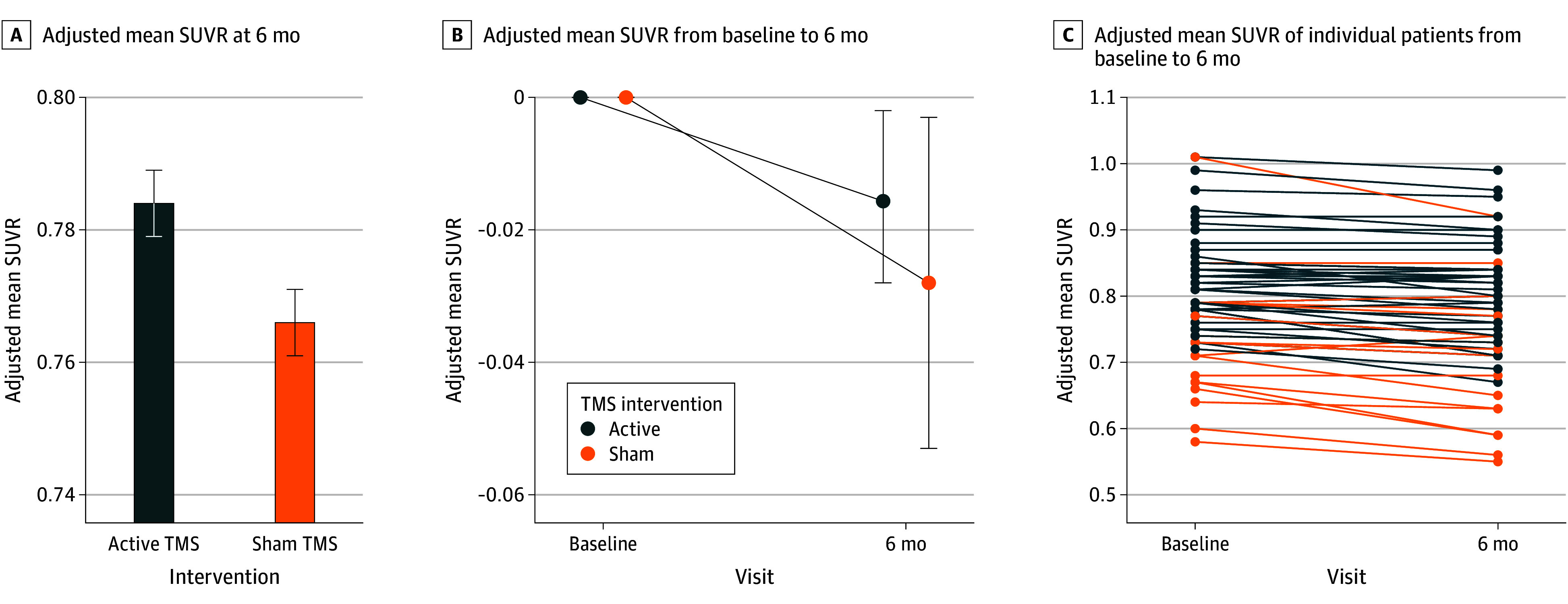
Comparison of Standardized Uptake Value Ratio (SUVR) Between Active and Sham Transcranial Magnetic Stimulation (TMS) Primary study outcome by treatment group. The analysis controlled for baseline SUVR, sex, and primary progressive aphasia variant. C, Each line represents a single participant. Error bars represent 95% CIs.

The adjusted mean SUVR at 6 months was 0.78 (95% CI, 0.77-0.79) for the active group and 0.77 (95% CI, 0.75-0.78) for the sham group (*P* = .046). The difference between groups was statistically significant (mean adjusted difference, 0.02 [95% CI, 0.01-0.03]; *F* = 4.17; *P* = .046; η^2^_p_ = 0.08), indicating less reduction in SUVR in the active treatment group compared with the sham group, while controlling for baseline SUVR, sex, and PPA variant (Figure 3 and eTable 6 in Supplement 2).

#### Secondary End Points

The adjusted mean MLSE at 6 months was 79.06 (95% CI, 76.48-81.64) for the active group and 71.35 (95% CI, 67.76-74.94) for the sham group. The mean adjusted difference was statistically significant (7.71 [95% CI, 3.06-12.35]; *F* = 11.07; *P* = .002; η^2^_p_ = 0.17), showing a worsening in MLSE in the sham group compared with the active group. There was also a significant mean adjusted difference on MLSE at 3 months (7.62 [95% CI, 1.67-13.56]; *F* = 6.61; *P* = .01; η^2^_p_ = 0.11). The adjusted mean MLSE at 3 months was 77.54 (95% CI, 74.22-80.87) for the active group and 69.92 (95% CI, 65.40-74.44) for the sham group (eTable 6 in Supplement 2).

The adjusted mean for confrontation naming of trained words at 6 months was 143.81 (95% CI, 137.26-150.35) for the active group and 119.99 (95% CI, 110.79-129.19) for the sham group. The mean adjusted difference was statistically significant (23.81 [95% CI, 11.85-35.77]; *F* = 15.97; *P* < .001; η^2^_p_ = 0.24), showing a favorable effect for the active group. The mean adjusted difference was also significant at 3 months (15.44 [95% CI, 4.81-26.08]; *F* = 8.59; *P* = .005; η^2^_p_ = 0.14). At 3 months, the adjusted mean of naming for the active group was 136.57 (95% CI, 130.71-142.44) and 121.77 (95% CI, 114.02-129.51) for the sham group (eTable 6 in Supplement 2).

The analysis did not reveal a significant mean adjusted difference on words per minute at 6 months (3.41 [95% CI, −8.86 to 15.68]; *F* = 0.32; *P* = .58; η^2^_p_ = 0.01) or 3 months (5.89 [95% CI, −3.58 to 15.36]; *F* = 1.57; *P* = .22; η^2^_p_ = 0.04). The adjusted mean for words per minute was 62.73 (95% CI, 55.94-69.52) for the active group and 59.32 (95% CI, 50.10-68.54) for the sham group at 6 months and 65.46 (95% CI, 60.19-70.74) for the active group and 59.57 (95% CI, 52.48-66.66) for the sham group at 3 months (eTable 6 in Supplement 2).

Regarding the functional impairment in daily living activities, the adjusted mean for IDDD at 6 months was 43.54 (95% CI, 40.61-46.48) for the active group and 48.94 (95% CI, 44.91-52.96) for the sham group, revealing a favorable effect for the active group (−5.39 [95% CI, −10.73 to −0.05]; *F* = 4.10; *P* = .048; η^2^_p_ = 0.07). The mean adjusted difference was not significant at 3 months (−1.81 [95% CI, −6.16 to 2.53]; *F* = 0.70; *P* = .41; η^2^_p_ = 0.01). The adjusted mean for IDDD at 3 months was 45.81 (95% CI, 43.35-48.27) for the active group and 47.62 (95% CI, 44.39-50.85) for the sham group (eTable 6 in Supplement 2). The analysis of the individual items for IDDD associated with language is shown in eTable 7 in Supplement 2.

The adjusted mean for NPI at 6 months was 6.99 (95% CI, 4.83-9.16) for the active group and 11.25 (95% CI, 7.98-14.53) for the sham group, showing greater worsening of the neuropsychiatric symptomatology in the sham group (−4.25 [95% CI, −8.37 to −0.14]; *F* = 4.30; *P* = .043; η^2^_p_ = 0.07). The effect was not significant at 3 months (−4.00 [95% CI, −0.82 to 0.27]; *F* = 3.51; *P* = .07; η^2^_p_ = 0.06). At 3 months, the adjusted mean for NPI was 7.38 (95% CI, 5.04-9.71) for the active group and 11.39 (95% CI, 8.05-14.73) for the sham group (eTable 6 in Supplement 2).

**Figure 4.**
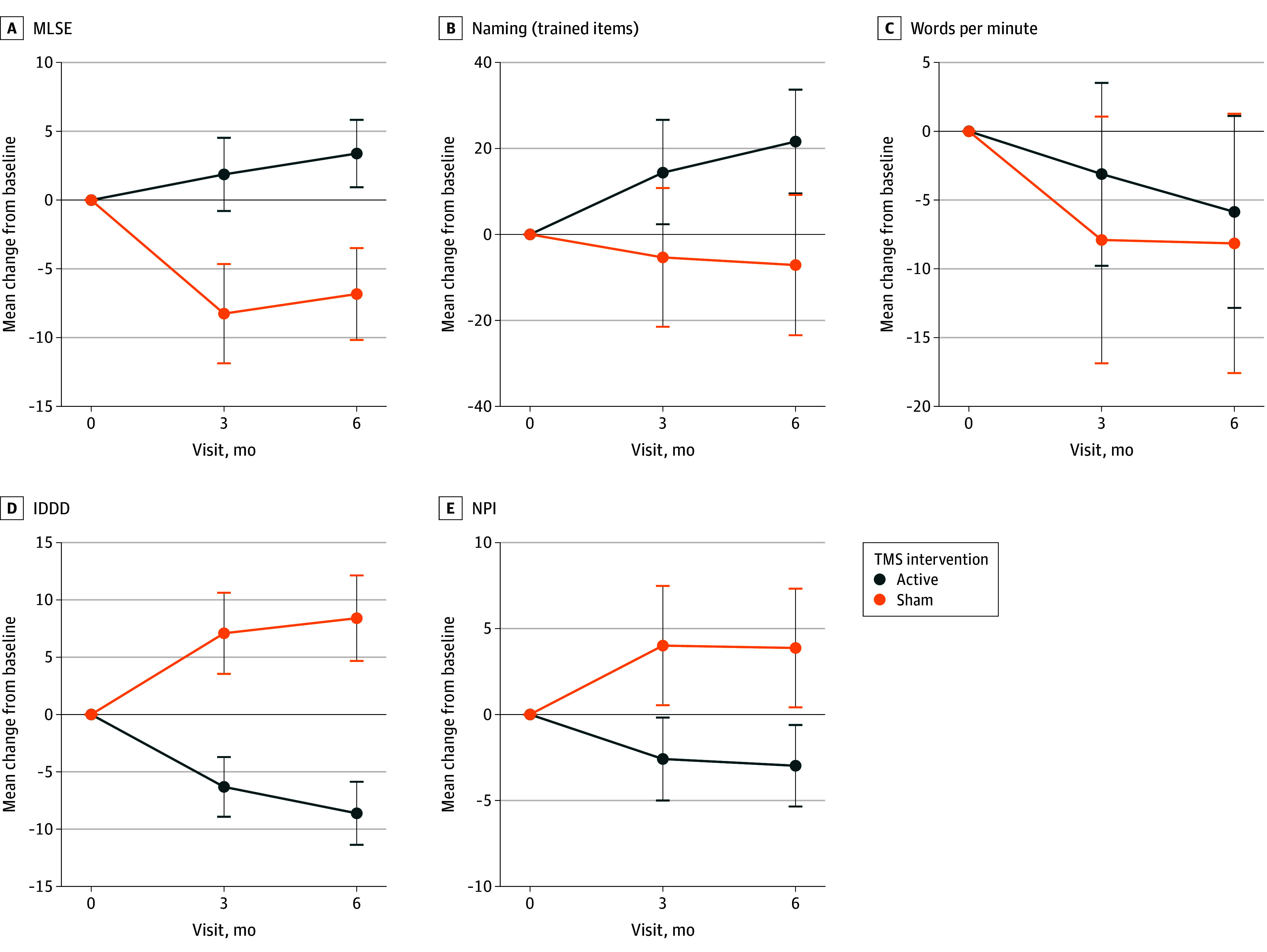
Mean Standardized Uptake Value Ratio (SUVR) Change From Baseline of the Secondary Outcomes by Treatment Group Error bars represent 95% CIs. IDDD indicates Interview for Deterioration of Daily Living in Dementia; MLSE, Mini Linguistic State Examination; NPI, Neuropsychiatric Inventory; TMS, transcranial magnetic stimulation.

Adjusted mean changes are represented in Figure 4; individual changes are shown in the eFigure in Supplement 2. Raw scores for each primary and secondary outcome are shown in eTable 8 in Supplement 2.

#### Safety

A total of 12 adverse events (AEs) were reported (eTable 9 in Supplement 2). In the sham-TMS group, 2 participants (9.5%) experienced serious AEs (diagnoses of cancer) that were unrelated to the treatment. They were withdrawn from the clinical trial when chemotherapy treatment began. In the active-TMS group, 1 mild AE (2.4%) was related to the TMS treatment (pain during TMS administration). This AE required modification of the TMS protocol, reducing the intensity during the initial sessions and achieving the predefined intensity after session 12.

Three participants (14.3%) in the sham group and 6 (14.4%) in the active group showed mild, unrelated AEs. There were no statistically significant differences between treatment groups for the number of AEs.

#### Treatment Adherence

The majority of participants (58 of 63 [92.1%]) adhered to the full treatment regimen, with 30 sessions (100%) completed as scheduled. There were 3 of 58 enrolled individuals (5.2%) who did not complete all 30 treatment sessions (1 active-TMS participant and 2 sham-TMS participants). No major deviations from the treatment protocol were reported.

## Discussion

In this randomized clinical trial, we found that TMS administered over a 6-month period improved or mitigated decline in regional brain metabolism, trained language abilities (ie, naming of trained objects), functional impairment, and neuropsychiatric symptoms in participants with PPA. Favorable outcomes have previously been reported for brain stimulation (both transcranial direct current stimulation and TMS) applied over a few weeks in people with PPA^23^; however, to our knowledge, this is the first trial to evaluate the effectiveness of TMS over a prolonged period of 6 months. A recent study also reported positive results from 6 months of TMS therapy targeting the precuneus in participants with AD.^24^ In our study, we selected the left DLPFC as the target for TMS, based on previously reported positive outcomes in nonfluent and semantic variants, as well as anecdotal cases in lvPPA.^15^ We used intermittent theta-burst stimulation, a protocol considered to be equally effective and safe as repetitive TMS^25^ but more practical due to its shorter application time. Notably, the brain stimulation protocol was combined with a language intervention specifically proven to be effective for individuals with PPA (especially semantic and logopenic variants), which engages semantic, phonologic, and orthographic systems and promotes the use of self-cueing strategies.^18,19,20,21^

In this context, the stability of naming performance on trained items in the sham group and the greater improvement observed in the active group suggest that TMS enhances the effects of language intervention in these participants. Given that individuals with PPA experience continual and inevitable progression of symptoms, these results suggest that TMS paired with evidence-based speech-language intervention may slow the progression of symptoms and associated brain changes. Conversely, we did not find significant differences in the assessment of words per minute in connected speech. However, the high variability of this parameter within and between groups makes it difficult to detect differences. Evaluating additional parameters derived from these samples of participants could help assess the potential impact of this intervention on connected speech. In addition, although our study was not powered to detect differences in the therapy across PPA variants, the specific analysis of each PPA variant in response to language therapy should also be of interest in future studies, with the aim of identifying the most effective protocols based on the baseline language characteristics of each participant.

Another important finding was the improvement in daily living activities and neuropsychiatric symptoms, suggesting that the therapy’s effects extend beyond trained language skills alone.^26^ Moreover, the therapy was safe, and adherence was very high, with only 2 withdrawals in the sham group due to causes unrelated to the study. Overall, these findings suggest that the combination of TMS and language therapy is a feasible and effective treatment option for PPA.

### Limitations

The current study has several limitations. First, we selected the left DLPFC as a common target for all participants, following our short-term findings in nfvPPA and svPPA.^27^ Further studies are needed to identify whether other targets could be even more effective and determine whether a personalized targeting approach (eg, target, dose, language training stimuli, and PPA type) would provide additional benefits. Second, there were baseline differences between the active and sham groups, including a trend toward higher representation of women in the active group and lower FDG-PET uptake in the sham group. To account for this, we controlled for sex and baseline SUVR in all analyses. Third, this was a single-center trial with a relatively homogeneous sample of participants from cultural and linguistic perspectives. Future research should include more diverse populations.^28^ Nevertheless, relatively few studies have been conducted in Spanish speakers with PPA, and this study represents 1 small step toward increasing our knowledge of the effects of intervention in languages that are less represented in the current literature. Fourth, although the trained words were tailored to each participant based on their naming performance, they were not selected by the participants themselves. Including a personally relevant vocabulary might have enhanced ecologic validity; however, individualized word selection helped ensure consistent targeting of language deficits while maintaining methodologic standardization. In this regard, we observed some positive effects on the language items of the IDDD, but the use of functional communication measures would be of interest in future studies to confirm the therapy’s impact on daily living.^29^ Finally, although active TMS was associated with favorable outcomes, including FDG-PET imaging findings potentially suggestive of neuroplastic effects, whether these changes are clinically meaningful remains unknown.

## Conclusions

In this randomized clinical trial of participants with PPA, intermittent theta-burst stimulation applied to the left DLPFC for 6 months combined with language therapy was associated with a reduction in decline of regional brain metabolism and improvements in trained language abilities, functional independence, and neuropsychiatric symptoms. Future studies should investigate the potential for TMS paired with an evidence-based speech-language intervention to sustain or extend these benefits beyond 6 months.

## Notes

Supplement 1.
Trial Protocol

Supplement 2. **eTable 1.** Summary of Naming Hierarchy for PPA **eTable 2.** Baseline Demographic and Clinical Characteristics (Per Protocol Analysis for the Primary Outcome) **eTable 3.** Mean (SE) of Cognitive and Language Assessment at Baseline **eTable 4.** Comparison Between Participants Who Completed and Did Not Complete the Treatment **eTable 5.** Comparison of Participants Who Underwent Complete Versus Incomplete Outcome Assessments **eTable 6.** Summary of Treatment Outcomes at 3 and 6 Months **eTable 7.** Summary of IDDD Language Outcomes at 3 and 6 Months **eTable 8.** Raw Scores for Primary and Secondary Outcomes at Baseline, 3 Months, and 6 Months **eTable 9.** Adverse Events **eFigure.** Individual Changes in the Secondary Outcomes **eAppendix.** Study Visit Procedures
eReferences

Supplement 3.
Data Sharing Statement
