# Error in Figure

**DOI:** 10.1001/jamanetworkopen.2025.48565

**Published:** 2025-11-19

In the Original Investigation titled “Long-Term Therapy With Transcranial Magnetic Stimulation in Primary Progressive Aphasia: A Randomized Clinical Trial,”^1^ published August 11, 2025, the y-axes in Figure 4A-E were incorrectly labeled “Mean SUVR change from baseline” instead of “Mean change from baseline.” This article has been corrected.
