# Supplementary material for: Long-Term Therapy With Transcranial Magnetic Stimulation in Primary Progressive Aphasia: A Randomized Clinical Trial
Source: JAMA Netw Open. 2025 Aug 11;8(8):e2526129. doi: 10.1001/jamanetworkopen.2025.26129 (PMC12340657; doi:10.1001/jamanetworkopen.2025.26129)
Supplement: Supplement 1. — Trial Protocol [file jamanetwopen-e2526129-s001.pdf]

Study protocol

Long-term Effect of Transcranial Magnetic Stimulation in Primary Progressive Aphasia (RECONNECT-PPA): a Randomized, Double-Blind Clinical Trial

ClinicalTrials-gov Identifier:

NCT05842473

1. STUDY

Clinical trial involving low-intervention therapies.

1.1. STUDY DESIGN

A prospective, randomized, controlled, double-blind, parallel-group clinical trial to evaluate the efficacy and safety of TMS combined with speech therapy compared to speech therapy alone on brain metabolism, language, and neuropsychiatric symptoms in patients with primary progressive aphasia.

1.2. DESCRIPTION OF STUDY INTERVENTIONS

This study proposes the use of transcranial magnetic stimulation (TMS) combined with speech therapy. Both treatments have previously been used with optimal short-term results (6 weeks).

1.3. NUMBER OF PATIENTS

60 patients.

1.4. SPONSOR INFORMATION

Dr. Jordi Matías-Guiu Antem

1.5. TIMELINE AND EXPECTED DURATION OF THE TRIAL

Each patient will undergo treatment for 6 months. However, given the prevalence of the disease, the trial is expected to last approximately 3 years.

2. STUDY JUSTIFICATION

Primary progressive aphasia (PPA) is a clinical syndrome characterized by an insidious decline in language abilities due to a neurodegenerative etiology. Language impairment must be the primary clinical manifestation for at least the first two years of progression, with other cognitive domains (memory, visuospatial skills, etc.) remaining intact, and limitations in daily life activities being solely attributable to language dysfunction. Described in the medical literature by Mesulam, it is a heterogeneous syndrome that can be the initial presentation of various neurodegenerative diseases.

Three main clinical subtypes of PPA have been described, each associated with a specific anatomical and functional pattern (Gorno-Tempini 2004, Bonner 2010). These variants include the agrammatic/non-fluent variant (nfvPPA), the semantic variant (svPPA), and the logopenic/phonological variant (lvPPA). The first two variants are typically grouped under frontotemporal degenerations, while the logopenic variant is often associated with Alzheimer's disease.

Currently, no treatment has been proven effective for PPA. No drug has demonstrated efficacy. Since the logopenic variant is an atypical form of Alzheimer's disease, the drugs approved for Alzheimer's, such as cholinesterase inhibitors and memantine, are considered for use, although their real effect remains unknown. At present, the only treatment shown to provide some benefit is speech therapy. However, in the absence of pharmacological treatments, many patients do not receive speech therapy due to a certain therapeutic nihilism surrounding the disease.

In recent years, significant advances have been made in the field of speech therapy, although most studies have implemented short-term treatments lasting only a few weeks. Given the frequent association of behavioral symptoms, especially in the semantic variant, the feasibility of long-term treatment remains unknown. Among the proposed therapies, word retrieval interventions have been the most studied (Henry et al., 2019; Volkmer et al., 2020; Crook et al., 2020).

In recent years, the potential utility of non-invasive neuromodulation therapies, particularly transcranial direct current stimulation (tDCS) and transcranial magnetic stimulation (TMS), has been explored in neurodegenerative diseases (Dong et al., 2020), and specifically in PPA (Norise & Hamilton, 2017; Nissim et al., 2020). Transcranial magnetic stimulation (TMS) is a technique based on the principle of electromagnetic induction to create an electric field in the brain. It is used as a non-invasive treatment for different conditions, such as depression, bipolar disorder, Parkinson's disease, epilepsy, and post-stroke aphasia rehabilitation.

Initial studies demonstrated that repetitive transcranial magnetic stimulation (rTMS) and/or transcranial direct current stimulation (tDCS) improved naming abilities in patients with Alzheimer's disease (Cotelli et al., 2006) and in patients with nfvPPA (Finocchiato et al., 2006; Cotelli et al., 2012; Trebastoni et al., 2013; Cotelli et al., 2014; Gervits et al., 2016) and svPPA (Teichmann et al., 2016).

Over the past four years, several studies have been published, most of which focus on transcranial direct current stimulation (Cotelli et al., 2020). Notably, the largest sample size study was conducted by Tsapkini and collaborators (2018), who randomized 36 patients with PPA to receive either tDCS (targeting the left inferior frontal gyrus) combined with speech therapy or sham tDCS combined with speech therapy. This was a crossover study involving 15 consecutive sessions over 3 weeks, followed by a 2-month washout period. The intervention showed favorable results for the treatment group, with benefits observed both in the trained items targeted by the speech therapy and in untrained items. The effect was especially beneficial for the non-fluent and logopenic variants. Subsequent

analyses of this trial revealed changes in brain connectivity (de Aguiar et al., 2020; Tao et al., 2021).

Table 1. tDCS studies in PPA (excluding studies with 5 or fewer patients and sub-studies of the main trial).

	Participants	Design	Neuromodulation	Sessions	Duration	Country	Findings
Cotelli et al. 2014	16 nfvPPA		tDCS (anode: DLPFC) and language therapy vs. sham-tDCS and language therapy	10	2 weeks	Italy	Greater improvement in trained items; no differences in untrained items
Gervits et al. 2016	2 nfvPPA<br>4 lvPPA	Pre-post	tDCS Frontotemporal region	10	2 weeks	USA	Improvement in language production
Teichmann et al. 2016	12 svPPA	Crossover	tDCS Anterior temporal region vs. sham	1	1 day	France	Improvement in semantic association tasks compared to sham
Roncero et al. 2017	6 nfvPPA<br>2 lvPPA<br>2svPPA	Crossover	tDCS inferior parietotemporal vs. sham	10	2 weeks	Canada	Improvement in naming for both trained and untrained items.
McConathey et al. 2017	6 nfvPPA<br>1lvPPA	Crossover	tDCS left frontotemporal vs. sham	10		USA	Improvement across various language elements, with differences in magnitude

							depending on initial deficit severity.
Tsapkini et al. 2018	14 nfvPPA<br>12 lvPPA<br>10 svPPA	Crossover	tDCS left inferior frontal gyrus vs. sham	15	3 weeks	USA	tDCS improvement in in naming/spelling, particularly in nfvPPA and lvPPA compared to sham
Roncero et al. 2019	12 PPA	Crossover	tDCS P3, F3, or sham	10	2 weeks	Canada	Improvement in naming, especially for trained items.

In a previous study, our group treated 20 patients with PPA (non-fluent and semantic variants) through 15 sessions of high-frequency repetitive TMS, observing clinical improvement in various linguistic tasks, a reduction in apathy, and an increase in cerebral metabolism in several brain regions primarily associated with language networks and the default mode network (Pytel et al., 2021). The TMS treatment protocol was selected based on a prior phase in which different protocols were tested (excitatory vs. inhibitory protocols, targeting different brain areas). It was concluded that excitatory protocols produced a more favorable effect, and different brain areas were selected as the most suitable (e.g., left inferior frontal gyrus, dorsolateral prefrontal cortex, etc.).

Table 2. TMS Studies in PPA (Excluding Studies with 5 or Fewer Patients)

	Participants	Design	Neuromodulation	Sessions	Duration	Country	Findings

Cotelli et al., 2012	10 nfvPPA	Pre-post	High-frequency rTMS on left DLPFC, right DLPFC, and sham	1	1 day	Italy	Improvement in naming
Antczak et al., 2018	11 (2 PPA, 9 bvFTD)	Pre-post	Bilateral high-frequency rTMS	10	2 weeks	Poland	Improvement in some cognitive tests and subjective impression
Margolis et al., 2019	8 PPA	-	rTMS on left DLPFC, right DLPFC, and sham coil	2	1 day	USA	-
Pytel et al., 2020	20 (14 nfvPPA, 6 svPPA)	Pre-post and crossover	High-frequency rTMS with personalized targeting and sham (vertex) with neuronavigation	15	4 weeks	Spain	Improvement in spontaneous speech, reduced apathy, and increased regional cerebral metabolism

TMS modulates cortical excitability and induces synaptic and non-synaptic changes (Monti et al., 2013). These changes can lead to modifications in cognition, neurotransmitters, and brain neuroplasticity (Rossi et al., 2009). Experimental studies have also suggested a potential neuroprotective effect (Caballero-Villarraso et al., 2021). The utility of TMS seems to be greater when combined with speech therapy, given the synergistic effect on brain plasticity. As previously reviewed and summarized in Tables 1-2, several studies have observed benefits of transcranial stimulation (either transcranial direct current or magnetic stimulation) in PPA, but to our knowledge, no studies have evaluated the long-term effects.

To date, there have been few clinical trials conducted in primary progressive aphasia (Reed et al., 2004; Kertesz et al., 2008; Johnson et al., 2010). One of the main challenges is the clinical heterogeneity of the syndrome, as there are three main variants, although additional subtypes are believed to exist. Additionally, it is a rare syndrome, which complicates recruitment. While language impairment is the primary clinical feature, the impairment is not uniform, both cross-sectionally and longitudinally. For example, confrontation naming is much more impaired in the semantic variant, followed by the logopenic variant and the non-fluent variant, where it tends to be more preserved. Furthermore, certain tasks may have a significant ceiling or floor effect in the disease. Recent documents suggest the use of advanced neuroimaging techniques, as they show a high correlation with the clinical status and are more sensitive and objective in detecting changes (Staffaroni et al., 2019; Boxer et al., 2020; Panza et al., 2020). In this regard, FDG-PET is a marker of synaptic dysfunction, a key element of the pathophysiology of neurodegenerative diseases. FDG-PET shows a high correlation with cognitive and linguistic function and the clinical stage of the disease, making it a valuable technique for monitoring. Moreover, the ability to perform quantitative or semi-quantitative analyses avoids inter-observer differences, increasing the reliability and reproducibility of the technique (Minoshima et al., 2021). Therefore, it is considered an appropriate technique to evaluate longitudinal changes in clinical trials (Bejanin et al., 2020).

3. HYPOTHESES

Primary Hypothesis

The treatment with TMS and speech therapy reduces disease progression in primary progressive aphasia, as determined by brain metabolism at 6 months, compared to speech therapy and sham TMS.

Secondary Hypotheses

The treatment with TMS and speech therapy improves naming ability following speech therapy, as determined by naming trained words, compared to speech therapy and sham TMS.

The treatment with TMS and speech therapy improves spontaneous speech, as determined by the number of words per minute, compared to speech therapy and sham TMS.

The treatment with TMS and speech therapy improves functional activity in daily life, as determined by the IDDD scale, compared to speech therapy and sham TMS.

The treatment with TMS and speech therapy improves the functional activity in primary progressive aphasia, as measured by the Mini-Linguistic State Examination (MLSE) at 6 months, compared to speech therapy and sham TMS.

The treatment with TMS and speech therapy reduces neuropsychiatric symptoms in primary progressive aphasia, as determined by the NPI scale at 6 months, compared to speech therapy and sham TMS.

4. STUDY OUTCOMES

4.1. Primary outcome

To compare the effect of TMS and speech therapy with speech therapy and sham TMS on the progression of primary progressive aphasia, as determined by brain metabolism on FDG-PET at 6 months.

4.2. Secondary outcomes

1. To compare the effect of TMS and speech therapy with speech therapy and sham TMS on spontaneous speech in primary progressive aphasia, as measured by the naming of trained words at 6 months.
2. To compare the effect of TMS and speech therapy with speech therapy and sham TMS on spontaneous speech in primary progressive aphasia, as measured by the number of words per minute at 6 months.
3. To compare the effect of TMS and speech therapy with speech therapy and sham TMS on functional activity in primary progressive aphasia, as measured by the IDDD scale (Interview for Deterioration in Daily Living Activities in Dementia) at 6 months.
4. To compare the effect of TMS and speech therapy with speech therapy and sham TMS on functional activity in primary progressive aphasia, as measured by the Mini-Linguistic State Examination (MLSE) at 6 months.
5. To compare the effect of TMS and speech therapy with speech therapy and sham TMS on neuropsychiatric symptoms in primary progressive aphasia, as measured by the Neuropsychiatric Inventory (NPI) at 6 months.

4.3. BIOMARKER SUBSTUDY

Substudies will be conducted with the following biomarkers, which will be voluntary and independent of each other. That is, a patient may choose not to participate in any of the substudies (MRI, blood biomarkers, EEG).

- Brain Magnetic Resonance Imaging (MRI): Participants will be offered the option to undergo brain MRI with the following sequences (3D-T1, 3D-FLAIR, ASL, DTI, and resting-state functional MRI) at 6 months.

- Serum Neurodegeneration Biomarkers: Serum samples will be collected at the baseline visit and 6 months to determine potential changes in serum biomarker levels.
- Electroencephalogram (EEG) (resting-state): The aim is to compare the effect of TMS combined with language therapy versus language therapy combined with sham TMS on the evolution of brain connectivity in primary progressive aphasia, assessed through resting-state EEG at 6 months.

5. SELECTION OF PATIENTS

5.1. STUDY POPULATION

Patients over 18 years of age diagnosed with primary progressive aphasia (PPA) according to the criteria established by Gorno-Tempini et al. (2011).

5.2 PATIENT INCLUSION CRITERIA

Eligible subjects for inclusion in this study must meet all of the following criteria:

1. Diagnosis of PPA according to the consensus criteria by Gorno-Tempini et al., supported by neuroimaging (PET-FDG and/or Magnetic Resonance Imaging).

2. Clinical Dementia Rating (CDR) score of 1 or less.

3. Language is the patient’s primary deficit.

4. Signed informed consent provided by the patient or their legal representative.

5.3 PATIENT EXCLUSION CRITERIA

Subjects meeting any of the following criteria are not eligible for inclusion in this study:

1. Patients diagnosed with a condition other than PPA that may cause language impairment.
2. History of epilepsy or evidence of focal epileptiform pathology on EEG recordings.
3. Presence of contraindications related to the treatments or procedures to be used (TMS and MRI), such as:
    a. Magnetically sensitive metal in the head or within 30.5 cm of the TMS coil that cannot be removed. Examples include aneurysm clips or coils, carotid or cerebral stents, implanted stimulators, electrodes, ferromagnetic implants in the ears or eyes, bullets or shrapnel fragments, and magnetically activated dental implants.

    b. Pregnancy.

4. Patients who are breastfeeding, pregnant, or planning to become pregnant within the next year. Fertile women must agree to use contraception throughout the study. If there is uncertainty at the time of inclusion, a pregnancy test will be conducted.
5. Patients with a terminal medical condition with a life expectancy of less than one year.
6. Patients with active malignant disease in the past two years.
7. Any condition that prevents participation or follow-up in the study.
8. Alcohol or substance abuse within the past year.
9. Major psychiatric disorders (e.g., schizophrenia, schizoaffective disorders, bipolar disorder, obsessive-compulsive disorders, or personality disorders).
10. Absolute inability to communicate (mutism) or poor language proficiency that prevents participation in the study, as determined by the investigator.
11. Severity of PPA that prevents the patient from following the study interventions or assessments at the time of inclusion.
12. Participation in another clinical trial within the past 4 months.
13. Chronic use of medications that could influence study outcomes:
    a. Antiepileptic drugs are permitted if on a stable dose for 3 months prior to inclusion. If required during the study due to a seizure episode, they may be added.
    b. Diazepam and derivatives are permitted only if on stable doses 3 months prior to inclusion. Adjustments are allowed during the study.
    c. Donepezil, Galantamine, Rivastigmine, and Memantine are permitted if on stable doses 3 months prior to inclusion.
    d. SSRIs (Selective Serotonin Reuptake Inhibitors) are permitted only if on stable doses 3 months prior to inclusion. They may be added if necessary during the study.
    e. Medications that can lower the seizure threshold (e.g., tricyclic antidepressants, antipsychotics) are permitted if on stable doses 3 months prior to inclusion. They may be added if necessary during the study.

5.4. WITHDRAWAL CRITERIA

All randomized patients will be included in the intention-to-treat analysis. The sponsor and/or investigator may withdraw any participant from the clinical trial if they meet any of the following withdrawal criteria:

1. Any condition that disrupts the study procedures.
2. Onset of severe depression.

3. The patient's own decision to withdraw.
4. Serious adverse events related to the treatment and study therapies.
5. Any cause resulting in the interruption of treatment for more than 4 weeks.
6. Patients undergoing chemotherapy.
7. Pregnancy.
8. Meeting any of the following exclusion criteria during the study:
    a. Presence of contraindications related to the treatments being used.
    b. Major psychiatric disorders (e.g., schizophrenia, schizoaffective disorders, bipolar disorder, or obsessive-compulsive disorders).
9. At the investigator's discretion and with confirmation from the sponsor.

5.5. WITHDRAWALS

In accordance with the Declaration of Helsinki and current legal regulations, participation in this study is entirely voluntary, and patients may withdraw from the study at any time without providing an explanation. If a patient requests withdrawal, the investigator must remove them from the study, document the withdrawal in the patient's medical record, and record it in the CRF.

5.6. MANAGEMENT OF WITHDRAWALS/DISCONTINUATIONS

After withdrawal from the study, patients will continue receiving standard clinical care for PPA. Withdrawn patients will not be replaced.

5.7. EARLY TERMINATION OF THE STUDY

The sponsor may decide to terminate the study in cases of unsatisfactory recruitment, safety concerns (e.g., the occurrence of adverse events of such incidence or severity as to pose a risk to patients' lives), or the emergence of new scientific information rendering the study no longer relevant.

6. DESCRIPTION OF THE TREATMENT

There will be two treatment arms:

1. Active TMS + speech therapy
2. Sham TMS + speech therapy

Patients will be randomized in a 2:1 ratio, stratified by PPA variant (non-fluent, semantic, logopenic).

7.1. TMS INTERVENTION

All patients included in the study will receive TMS and speech therapy.

- TMS: TMS is a technique for electrical stimulation of brain tissue through the generation of a magnetic field, modulating neuronal activity at the

stimulation site and interconnected neural networks. The study will use transcranial magnetic stimulation (TMS) delivered with a figure-of-eight coil guided by neuronavigation. An intermittent theta-burst stimulation (iTBS) protocol will be applied, following current clinical practice guidelines and recommendations for TMS (Lefaucheur et al., 2020). The theta-burst protocol allows for shorter stimulation times (<10 minutes per session) with effects comparable to traditional repetitive stimulation protocols. Neuronavigation, based on the patient's own MRI, ensures accurate targeting of the stimulation area, reducing inter- and intra-individual variability. Treatment will be applied to the left dorsolateral prefrontal cortex. Each patient will receive 10 consecutive sessions during the first 2 weeks, followed by 1 weekly session until completing 6 months of treatment.

- Sham TMS: For the sham TMS group, a sham coil indistinguishable from the therapeutic coil will be used. Sessions will occur at the same frequency as the active TMS group, ensuring that patients remain unaware of whether they are receiving sham or standard TMS. Only the staff responsible for administering the TMS will know the treatment assignment.
- Blinding: The evaluator conducting follow-up assessments will be blinded to the treatment allocation (active or sham TMS). Only the technician applying the TMS will know whether the treatment is active or sham.
- If a patient misses a scheduled TMS or speech therapy session, it will be rescheduled, preferably within the same week. If the treatment cannot be performed that week, the missed sessions will be scheduled for the following weeks (with one additional session per week). The maximum period without treatment sessions will be 4 weeks. If this period is exceeded, the patient will be withdrawn from the study.

7.2 SPEECH THERAPY INTERVENTION

We will use a treatment previously shown to be effective in PPA, which incorporates a modified hierarchy of steps from the Arizona Naming Cascade (Grasso et al., under review; Henry et al., 2013; 2019; Kim, 2017).

The treatment focuses on naming through guided retrieval of residual semantic, phonological, and orthographic information, with the aim of retraining specific vocabulary and teaching strategies to facilitate broader word retrieval. These strategies include semantic circumlocution and using knowledge of word form for self-cuing.

Table 3. Summary of Naming Hierarchy for PPA

Semantic Feature Analysis	Prompt semantic description with “Tell me about it.”<br><br>Summary of the description<br><br>Recall the picture
Orthographic /Phonemic Self-Cue	Written production of the target word, “Can you write the word?”<br><br>“Can you say the first sound?”
Written	Request repeated copy of written word (x3)
Semantic plausibility judgments	Ask yes/no questions regarding semantic features of target (x5)
Recall	Ask for recall of two semantic features plus spoken/written name<br><br>Ask for name and write the picture
Description of a complex picture	Provide a complex image with the target included in the scene<br><br>“Describe the picture using the word we are practicing”

During the first 2 weeks, the patient will receive 4 speech therapy sessions. Subsequently, each patient will receive 1 session per week. The speech therapy sessions will take place immediately after the TMS session.

8. CONCOMITANT MEDICATION

There is no medication incompatible with this clinical trial.

9. TREATMENT ALLOCATION

Patient inclusion in the study will be consecutive. A prospective record will be maintained for all subjects who are not included in the study, specifying the reason for exclusion. All patients aged 18 or older diagnosed with PPA according to the 2011 consensus criteria by Gorno-Tempini et al., who meet the inclusion criteria, do not meet any exclusion criteria, and have signed the informed consent form, will be invited to participate.

Once patients are included in the study, they will be randomized to one of the treatment arms using computerized randomization. Each patient will be assigned a unique numeric code for identification. Randomization will be stratified by the clinical subtype of PPA (non-fluent, semantic, and logopenic).

If a patient meets the study's eligibility criteria, the investigator will provide detailed information about the study, addressing all questions and concerns. The patient will then be given the informed consent form to review at their convenience and discuss with family or friends if necessary. If the patient agrees to participate, both the patient and the informing investigator will sign two copies of the consent form. One copy will be given to the patient, and the other will be filed in the investigator's study archive.

Once the patient is enrolled in the study, the investigator will log into the study's electronic CRF using their personal credentials to complete the inclusion process and perform the randomization. At this stage, the patient will be assigned a unique code for the study and allocated to one of the treatment arms.

10. BLINDING

The study is designed as a double-blind clinical trial, the patient will remain unaware of the neuromodulation treatment being received at any time (active TMS or sham TMS).

11. UNBLINDING AND EMERGENCY PROCEDURES

Unblinding will only occur if the patient experiences an emergency where knowledge of the study treatment received is necessary to guide medical decisions.

If a patient meets the criteria for unblinding, the PI may access the treatment allocation by completing the "Unblinding" form in the electronic case report form (eCRF).

12. DATA COLLECTION AND MONITORING

I. Baseline Visit

At the Cognitive Neurology Clinic of Hospital Clínico San Carlos, patients who meet the eligibility criteria will receive both verbal and written information about the study. They will have the opportunity to ask any questions, as well as consult with their family and/or friends regarding their decision to participate.

If the patient decides to participate, two copies of the informed consent form will be signed—one will be given to the patient, and the other will be filed in the investigator's study archive. Participation in the study will also be documented in the patient's medical record, including the date of inclusion, the version of the signed consent form, and confirmation that a copy was provided to the patient.

The patient will be assigned a unique study code and randomized via the eCRF system.

During the Baseline Visit, the following activities will be performed:

- ✓ Evaluation of inclusion and exclusion criteria.
- ✓ Delivery of the patient information sheet and informed consent form.
- ✓ Randomization.
- ✓ Clinical Information: Date of birth, sex, years of formal education, year of PPA diagnosis, and relevant medical history.
- ✓ Concomitant Medication: Details of current medications.

The patient will be provided with a patient diary to record adverse events, visits to other specialists, and/or any required healthcare services. This diary is intended to assist the patient in adhering to treatment routines and facilitate study participation. The information recorded in the diary will be verified by a member of the research team and documented in the patient's medical records and eCRF during study visits.

After this visit, treatment appointments will be scheduled to ensure compliance with the established timeframes. The treatment initiation (corresponding to the relevant phase) must occur within 30 days following Visit 1 (Baseline Visit).

II. Visit 1 (Baseline Visit, Before Initiating the Intervention)

- ✓ During the baseline visit, the patient will undergo evaluations according to the following activities:
- ✓ Demographic Data
- ✓ Language and General Cognitive Evaluation
- ✓ MLSE (Mini Linguistic State Examination)
- ✓ Trained word naming
- ✓ Spontaneous speech (words per minute)
- ✓ IDDD (Interview for deterioration in Daily living Activities in Dementia)
- ✓ NPI (Neuropsychiatric Inventory)
- ✓ Patient Diary Review
- ✓ Concomitant Medication Review
- ✓ PET-FDG (Positron Emission Tomography with Fluorodeoxyglucose)

The visit may be conducted over a maximum of three days to prevent patient fatigue.

Language and general cognitive assessments may also be conducted within the three months preceding the start of treatment.

III. Intermediate Visit (+3 months after the start of the intervention)

During this visit, the patient will be evaluated following this schedule of activities:

- ✓ MLSE
- ✓ Trained Word Naming
- ✓ Spontaneous Speech (Words per Minute)
- ✓ IDDD
- ✓ NPI
- ✓ Patient Diary Review
- ✓ Concomitant Medication Review

IV. Final Visit (+6 months after the start of the intervention)

During the final visit, the patient will be evaluated following this schedule of activities:

- ✓ MLSE
- ✓ Trained Word Naming
- ✓ Spontaneous Speech (Words per Minute)
- ✓ IDDD
- ✓ NPI
- ✓ Patient Diary Review
- ✓ Concomitant Medication Review
- ✓ PET-FDG

The visit may be conducted over a maximum of three days to prevent patient fatigue.

The assessments will be completed within the following 15 days after the last treatment session.

13. ASSESSMENTS

13.1. Baseline Visit

- Age

- Sex

- Years of formal education

- Languages spoken and age of acquisition

- Mother tongue

- Laterality

- Family history of neurodegenerative diseases

- Duration of symptom

- Language assessment: Different tasks that assess language domains: Cookie Theft picture; Paradis narrative sequence picture; reading narrative text, words, capitalized words, foreign words, and non-words; omission of initial phoneme; spelling words; repetition of words, non-words, syllables and sentences; semantic association; naming pictures and actions; word-picture matching; synonyms; picture-action matching; orophonatory praxis; verb tense agreement; sentence comprehension.

- Cognitive assessment: Test included in NEURONORMA protocol (Peña-Casanova et al., 2009): memory span (forward and backward digit spans), the Corsi block-tapping test, Trail Making Test (TMT), Symbol Digit Modalities Test (SDMT), Visual Object and Space Perception Battery (VOSP; subtests for object decision, progressive silhouettes, position discrimination, and number location), Judgement of Line Orientation (JLO), Tower of London-Drexel University, Rey–Osterrieth Complex Figure Test (copy and memory), and Stroop Color–Word Interference Test.

- Addenbrooke's cognitive examination III (ACE-III): ACE-III is a general cognitive screening tool that assesses attention (temporal spatial orientation, 3-item registration, serial subtractions) memory (recall of 3 items, anterograde memory of a name and address, retrograde memory, and recall and recognition of a name and address) verbal fluency (words beginning with "p" and animals), language (comprehension of a complex command, sentence writing, single word repetition, proverb repetition, a 10-item confrontation naming task, a semantic task, and reading) and visuospatial skills (copy of intersecting infinity loops, copy of a cube, drawing of a clock, counting dots, and identifying incomplete letters).

13.2. Visit 1, 2 and 3 (week 0, week 12 and week 24)

- Mini-Linguistic state examination (MLSE)

The MLSE is a brief screening tool designed to assess language impairments in various neurodegenerative disorders, particularly in PPA. It includes a series of tasks that evaluate different language processes: naming, syllable repetition, repeat and point, non-word repetition, semantic association, sentence comprehension, reading, sentence repetition, writing, and picture description. These tasks are grouped in five language domains: motor, phonology, semantics, syntax, and working memory, and scoring is done by classifying the errors made by the subject. The time of administration is approximately 20 min.

- Naming of trained words

A list of 261 words from 8 different semantic categories: animals, food, objects, places, furniture, clothing, transportation, and body parts. Two consecutive sessions of oral naming of the list were conducted.

- Words per minute in spontaneous speech

The patient was shown a wordless book (Frog stories) and was asked to describe the story in detail for 3 minutes. The number of words per minute was calculated.

- Interview for Deterioration in Daily Life in Dementia (IDDD)

IDDD is a scale designed to assess functional decline in daily and instrumental activities. It is a caregiver-administered tool.

- Neuropsychiatric Inventory (NPI)

It is a caregiver-administered tool designed to assess neuropsychiatric symptoms. The NPI assesses 12 different symptom domains, which include: delusions, hallucinations, agitation/aggression, dysphoria/depression, anxiety, euphoria/elation, apathy/indifference, disinhibition, irritability/lability, aberrant motor behaviors, night-time behavioral disturbances and appetite/eating disturbances.

13.3. Visit 1 and 3 (week 0 and week 24)

The participants underwent an FDG-PET at the beginning and end of the study.

FDG-PET studies were performed using a Siemens Biograph TruePoint PET-CT scanner, which integrated a state-of-the-art detector with lutetium oxyorthosilicate crystals. Patients fasted for at least 6 hours prior to the scan. The dose was administered 30 minutes before image acquisition. Current European recommendations for the acquisition of FDG-PET brain studies were followed (Varrone et al. 2009).

FDG-PET images were pre-processed using the Statistical Parametric Mapping 12 program (Wellcome Trust Centre for Neuroimaging, Institute of Neurology, London). The analysis of cerebral metabolism was carried out using a broad region of interest that included much of the left hemisphere. This region of interest was obtained by comparing, through voxel-based analysis, a cohort of 70 PPA patients evaluated at two time points, and it was the region of interest used for sample size calculation. The relative uptake in this area was calculated at baseline and after 6 months of the study.

14. EFFICACY OUTCOMES

- **Primary outcome**

The primary variable of the study for the assessment of efficacy is the change in regional cerebral metabolism between the baseline and final visits.

- **Secondary outcomes**

MLSE

Trained Word Naming

Spontaneous Speech: number of words per minute

Activities of Daily Living: IDDD

Neuropsychiatric Symptoms: NPI

Adverse events related to treatments during the 6 months following randomization.

15. SAFETY MEASURES AND RISK MANAGEMENT

It is the responsibility of the investigator to detect and document any event that meets the criteria and definitions of an adverse event or serious adverse event as established in this protocol. Safety assessments will be conducted in accordance with Good Clinical Practice guidelines and current legislation. Adverse effects will be recorded during each study visit. The analysis will include the type of adverse effect and its frequency.

15.1. Definitions

15.1.1. Adverse Event (AE)

Any harmful health incident in a patient or clinical trial subject treated with a medication, regardless of whether there is a causal relationship with the treatment. An AE can therefore be any unfavorable and unintended sign (including an abnormal laboratory finding), symptom, or disease temporally associated with the use of an investigational drug, whether or not it is related to the drug.

15.1.2. Adverse Reaction (AR)

Any harmful and unintended reaction to an investigational drug, regardless of the dose administered. In this case, there is a suspected causal relationship between the investigational drug and the adverse event.

15.1.3. Serious Adverse Event (SAE) and Serious Adverse Reaction (SAR)

Any adverse event or adverse reaction that, at any dose: results in death, is life-threatening, requires hospitalization or prolongs an existing hospitalization,

causes permanent or significant disability or incapacity, or leads to a congenital anomaly or birth defect. For reporting purposes, any suspected AE or AR considered medically significant will also be treated as serious, even if it does not meet the above criteria. This includes medically significant events requiring intervention to prevent one of the outcomes. Additionally, any suspected transmission of an infectious agent through a medication will be reported as serious.

15.1.4. Unexpected Adverse Reaction (UAR):

Any AR whose nature, intensity, or consequences do not align with the reference information for the medication (e.g., the investigator’s brochure for an investigational drug not yet authorized for marketing, or the product’s data sheet in the case of an authorized medication).

15.2. Causality Assessment Criteria

The causal relationship between the investigational product and the occurrence of an AE will be established based on clinical judgment. Other potential causes, such as the natural history of underlying diseases, concomitant treatments, other risk factors, and the temporal relationship of the event to the investigational product, will be considered and evaluated.

The causality of an AE with the investigational medication will be determined based on the following definitions:

- **Related AE:** The temporal relationship of the AE with the investigational product suggests a possible causal relationship, and the event cannot be explained by factors such as the patient's clinical condition or therapeutic interventions.

- **Unrelated AE:** The temporal relationship of the AE with the investigational product suggests an improbable causal relationship, or other factors (such as concomitant medications, conditions, or other therapeutic interventions) provide a satisfactory explanation for the AE.

The investigator must assess the causality of all adverse events (serious or not) and report this assessment in accordance with the requirements for reporting serious adverse events, if applicable.

If the investigator is uncertain about the causality, for reporting purposes, the adverse event will be considered related to the investigational product.

15.3. Intensity Assignment

For all observed AEs, the investigator will use the following definitions to classify the intensity of any AR recorded during the study in the CRF:

- **Mild**: Does not cause limitations in daily activities.
- **Moderate**: Causes some limitation in performing daily activities.
- **Severe**: Prevents the performance of daily activities.

15.4. Expectedness Assessment

An AE or AR is considered unexpected if the experience (in terms of nature, severity, or frequency) is not described in the reference document for the investigational product, which in this case is TMS.

15.5. Procedure for AE Recording

The recording of AEs must be carried out by the study's investigative team. The investigator will systematically monitor and document all reported adverse events in the clinical records, whether spontaneously reported by the patient or identified during study visits. This monitoring will occur from randomization until the final follow-up visit for each subject.

Only ARs and all SAEs/SARs will be recorded in the CRF for each subject. For these events, the investigator must specify the start date, causality, intensity, severity, treatment, and outcome. If treatment is administered for any of these events, it must also be recorded in the CRF.

All AEs will be followed until resolution, stabilization, or determination that the investigational treatment or participation in the study was not the cause. Likewise, follow-up will be conducted for any AEs persisting after the study has concluded until their final outcome is determined.

Analytical abnormalities or other evaluations (e.g., vital signs) detected during the trial, or present at the time of signing the informed consent and worsening after the trial begins, will only be recorded as specified in this protocol if they are considered clinically significant by the investigator or related to the investigational medication.

15.6. SAE Reporting Procedure

Investigators must immediately notify the Principal Investigator (always within 24 hours of becoming aware) of all SAEs using the designated form (SAE Reporting Form).

16. STATISTICAL ANALYSIS

16.1. DESCRIPTION OF THE STATISTICAL ANALYSIS

All statistical analysis will be performed by intention-to-treat. The safety analysis will include all patients who have received at least one dose of treatment or one cycle of TMS.

For the analysis of normality and equality of variances of continuous variables, the Shapiro-Wilk test and Levene's test will be used, respectively. Quantitative variables will be presented as mean and standard deviation and will be compared using the Student's t-test or non-parametric test where appropriate. Qualitative variables will be expressed as frequency and percentage and compared using the $\chi 2$ test and Fisher's exact test when at least 25% of the cells in the contingency table show an absolute frequency of less than 5.

The primary analysis will be the difference in brain metabolism in the region of interest involved in the disease between the two treatment arms at 6 months. A statistical analysis will be used with a p-value <0.05 (two-tailed). The same analysis will be used to evaluate the change in the secondary outcomes of the study.

15.2. SAMPLE SIZE CALCULATION

The sample size calculation was based on the primary endpoint. In a sample of 70 patients with PPA who were longitudinally evaluated with two FDG-PET studies, the rate of decline in brain metabolism in a region of interest covering a large part of the left hemisphere follows a linear regression: $y = 74.82 + 15.16 * \text{number of months}$. This indicates a reduction in brain metabolism at 6 months of 167.78 (122–206). Assuming that the treatment could reduce this decline in brain metabolism by at least 20%, the estimated required sample size is 54 participants. Considering a 10% dropout rate, the final sample size was established as 60 participants.

17. ETHICAL AND LEGAL ASPECTS

17.1. APPLICABLE LEGISLATION

The study will comply with Good Clinical Practice (GCP) ICH guidelines and the current legislation on biomedical research (Biomedical Research Act 14/2007, BOE 4-VII-2007) and clinical trials development (Royal Decree 1090/2015).

17.2. CONFIDENTIALITY

The investigator will be responsible for keeping the necessary information about each patient (initials of the name, family members' phone number, social security number, and identity in the study) so that health authorities can access this

information if necessary, while maintaining the subject's name confidential. This information must be kept confidential for as long as legally stipulated, according to the legislation outlined in the General Data Protection Regulation.

Confidentiality of the study participants will be maintained at all times. The data collected for the study will be identified by a code, and only the investigator and collaborators, as well as the monitors, will be able to link the data to the patient and their medical history. Therefore, the patient’s identity will not be revealed to anyone, except in the following cases:

a. Authorized personnel by the sponsor, when necessary, to verify the study data and procedures, but always maintaining confidentiality in accordance with the current legislation;
b. In the event of a medical emergency;
c. Legal requirement by health authorities.

The data from this study will be used only for its specific purposes.

17.3. CIVIL LIABILITY INSURANCE

As described in Royal Decree 1090/2015, the CEIm (Ethics Committees for Investigation with medicinal products) is requested to exempt the obligation of an insurance policy for this clinical trial since it is a low-intervention clinical trial, employing authorized research interventions with indications stated in the technical data sheet of the equipment. Furthermore, there are no diagnostic or follow-up procedures that would pose additional risks compared to routine clinical practice.

17.4. PATIENT INFORMATION SHEET AND INFORMED CONSENT

It is the responsibility of the investigator or designated person to obtain written informed consent from the participating patients after adequately explaining the study's objectives, methods, expected benefits, and potential risks.

The investigator or delegated person must emphasize to the subjects that they are completely free to decline participation or withdraw at any time and for any reason without it affecting their medical care.

The informed consent of the subject will be documented in the eCRF in a dedicated section.

17.5. DATA RECORDING

The research data will be collected in an electronic Case Report Database (eCRF) specifically designed for this project.

17.6. PUBLICATION CONDITIONS

The results of this clinical trial will be communicated at scientific meetings and published in scientific journals.

17.7. COMPENSATION

There is no compensation for the investigators and sub-investigators of this study. No payments are planned for the patients participating in the study.

18. REFERENCES

-Antczak J, Kowalska K, Klimkowicz-Mrowiec A, Wach B, Kasprzyk K, Banach M, Rzeznicka-Brzegowy K, Kubica J, Słowik, A (2018) Repetitive transcranial magnetic stimulation for the treatment of cognitive impairment in frontotemporal dementia: an open-label pilot study. *Neuropsychiatr Dis Treat* 14, 749.

-Bejanin A, Tammewar G, Marx G, Cobigo Y, Iaccarino L, Kornak J, Staffaroni AM, Dickerson BC, Boeve BF, Knopman DS, Gorno-Tempini ML, Miller BL, Jagust WJ, Boxer AL, Rosen HJ, Rabinovici GD. Longitudinal structural and metabolic changes in frontotemporal dementia. Neurology 2020; 95:e140-154.

-Boxer AL, Gold M, Feldman H, Boeve BF, Dickinson SLJ, Fillit H, Ho C, et al. New directions in clinical trials for frontotemporal lobal degeneration: methods and outcome measures. Alzheimers Dement 2020; 16:131-143.

-Caballero-Villarraso J, Medina FJ, Escribano BM, Agüera E, Santamaria A, Pascual- Leone A, Túnez I. Mechanisms involved in neuroprotective effects of transcranial magnetic stimulation. CNS Neurol Disord Drug Targets 2021. Doi:10.2174/ 1871527320666210809121922

-Cotelli M, Manenti R, Cappa SF, Geroldi C, Zanetti O, Rossini PM, et al. Effect of transcranial magnetic stimulation on action naming in patients with Alzheimer disease. Arch Neurol 2006; 63:1602-1604.

-Cotelli M, Manenti R, Alberici A, Brambilla M, Cosseddu M, Zanetti O, Miozzo A, Padovani A, Miniussi C, Borroni B. Prefrontal cortex rTMS enhances action naming in progressive non-fluent aphasia. Eur J Neurol 2012; 19:1404-1412.

-Cotelli M, Manenti R, Petesi M, Brambilla M, Cosseddu M, Zanetti O, Miniussi C, Padovani A, Borroni B. Treatment of primary progressive aphasias by transcranial direct current stimulation combined with language. J Alzheimers Dis 2014; 39:799-808.

-Cotelli M, Manenti R, Ferrari C, Gobbi , Macis A, Cappa SF. Effectivness of language training and non-invasive brain stimulation on oral and written naming performance in primary progressive aphasia: a meta-analysis and systematic review. Neurosci Biobehav Rev 2020;108:498-525.

-Croot K, Raiser T, Taylor-Rubin C, et al. Lexical retrieval treatment in primary progressive aphasia: An investigation of treatment duration in a heterogeneous case series. Cortex 2019;115:133–58.

- Cummings JL, Mega M, Gray K, Rosenberg-Thompson S, Carusi DA, Gornbein J. The Neuropsychiatric Inventory: comprehensive assessment of psychopathology in dementia. *Neurology* 1994; 44: 2308–2314

-de Aguiar V, Zhao Y, Faria A, Ficek B, Webster K T, Wendt H, Wang Z, Hillis AE, Onyike CU, Frangakis C, Caffo B, Tsapkini K (2020) Brain volumes as predictors of tDCS effects in primary progressive aphasia. *Brain Lang* 200, 104707.

- Dong X, Yan L, Huang L, Guan X, Dong C, Tao H, Wang T, Qin X, Wan Q (2018) Repetitive transcranial magnetic stimulation for the treatment of Alzheimer's disease: A systematic review and meta-analysis of randomized controlled trials. *PloS one* 13, e0205704

-Finocchiato C, Maimone M, Brighina F, Piccoli T, Giglia G, Fierro B. A case study of primary progressive aphasia: improvement on verbs after rTMS treatment. Neurocase 2006; 12:317-321.

-Gervits F, Ash S, Branch Coslett H, Rascovsky K, Grossman M, Hamilton R. Transcranial direct current stimulation for the treatment of primary progressive aphasia: an open-label pilot study. Brain Lang 2016;162:35-41.

- Gorno-Tempini ML, Hillis AE, Weintraub S, Kertesz A, Mendez M, Cappa SF, Ogar JM, Rohrer JD, Black S, Boeve BF, Manes F, Dronkers NF, Vandenberghe R, Rascovsky K, Patterson K, Miller BL, Knopman DS, Hodges JR, Mesulam MM, Grossman M. Classification of primary progressive aphasia and its variants.

Neurology 2011;76:1006-1014.

-Henry ML, Hubbard HI, Grasso SM, et al. Treatment for word retrieval in semantic and logopenic variants of primary progressive aphasia: Immediate and long-term outcomes. J Speech Lang Hear 2019;62:2723-2749.

-Johnson NA, Rademaker A, Weintraub S, Gitelman D, Wienecke C, Mesulam M. Pilot trial of memantine in primary progressive aphasia. Alzheimers Dis Assoc Disord 2010;24:308.

-Kertesz A, Morlog D, Light M, Blair M, Davidson W, Jesso S, Brashear R. Galantamine in frontotemporal dementia and primary progressive aphasia. Dement Geriatr Cogn Disord 2008;25:178-185.

- Lefaucheur JP, Aleman A, Baeken C, Benninger DH, Brunelin J, Di Lazzaro V, Filipovic SR, Grefkes C, Hasan A, Hummel FC, Jääskeläinen SK, Langguth B, Leocani L, Londero A, Nardone R, Nguyen JP, Nyffeler T, Oliveira-aia AJ, Oliveiro A, Padberg F, Palm U, Paulus W, Poulet , Quartarone A, Rachid F, Rektorova I, Rossi S, Sahlsten H, Schecklmann M, Szeleky D, Ziemann U (2020) Evidence-based guidelines on the therapeutic use of repetitive transcranial magnetic stimulation (rTMS): an update (2014–2018). *Clinical neurophysiol* 2020;131: 474-528

-Margolis SA, Festa EK, Papandonatos GD, Korthauer LE, Gonsalves MA, Oberman L, Heindel WC, Ott BR (2019) A pilot study of repetitive transcranial magnetic stimulation in primary progressive aphasia. *Brain Stimul* 12, 1340–1342.

-Minoshima S, Mosci K, Cross D, Thientuyakit T. Brain [F-18]FDG PET for clinical dementia workup: differential diagnosis of Alzheimer's disease and other types of dementing disorders. Semin Nucl Med 2021;51:230-240.

- Norise Catherine , Hamilton Roy H. Non-invasive Brain Stimulation in the Treatment of Post-stroke and Neurodegenerative Aphasia: Parallels, Differences, and Lessons Learned. Frontiers in Human Neuroscience 2017; 10:675.

-Nissim NR, Moberg PJ, Hamilton RH. Efficacy of noninvasive brain stimulation (tDCS or TMS) paired with language therapy in the treatment of primary progressive aphasia: an exploratory meta-analysis. Brain Sci 2020;10:597.

-Panza F, Lozupone M, Seripa D, Daniele A, Watling M, Giannelli G, Imbimbo BP. Development of disease-modifying drugs for frontotemporal dementia spectrum disorders. Nat Rev Neurol 2020;16:213-228.

-Reed DA, Johnson NA, Thompson C, Weintraub S, Mesulam M. A clinical trial of bromocriptine for treatment of primary progressive aphasia. Ann Neurol 2004;56:750.

-Rossi S, Hallet M, Rossini PM, Pascual-Leone A, and The Safety of TMS Consensus Group. Safety, ethical considerations, and application guidelines for the use of transcranial magnetic stimulation in clinical practice and research. Clin Neurophysiol 2009; 120:2008-2039.

-Staffaroni AM, Ljubenkov PA, Kornak J, Cobigo Y, Datta S, Marx G et al. Longitudinal multimodal neuroimaging and clinical endpoints for frontotemporal dementia clinical trials. Brain 2019;142:443-459.

-Tao Y, Ficek B, Wang Z, Rapp B, Tsapkini K. Selective functional network changes following tDCS-Augmented Language Treatment in Primary Progressive Aphasia. Front Aging Neurosci 2021;13:681043.

-Teichmann M, Lesoil C, Godard J, Vernet M, Bertrand A, Levy R, Dubois B, Lemoine L, Truong DQ, Bikson M, Kas A, Valero-Cabré A. Direct current stimulation over the anterior temporal áreas boosts semantic processing in primary progressive aphasia. Ann Neurol 2016;80:693-707.

-Trebbastoni A, Raccah R, de Lena C, Zangen A, Inghilleri M. Repetitive deep transcranial magnetic stimulation improves verbal fluency and written language in a patient with primary progressive aphasia-logopenic variant (LPPA). Brain Stimul 2013; 6:545-553.

- Tsapkini K, Webster KT, Ficek BN, Desmond JE, Onyike CU, Rapp B, Frangakis C, Hillis AE (2018) Electrical brain stimulation in different variants of primary progressive aphasia: A randomized clinical trial. Alzheimers Dement (N Y) 4, 461–472

- Varrone A, Asenbaum S, Vander Borght T, Booij J, Nobili F, Någren K, Darcourt J, Kapucu OL, Tatsch K, Bartenstein P, Van Laere K (2009) EANM procedure guidelines for PET brain imaging using [18 F] FDG, version 2. Eur J Nucl Med Mol Imaging 2009; 36: 2103-2110.

-Volkmer A, Rogalski , Henry M, Taylor-Rubin C, Ruggero L, Khayum R, Kindell J, Gorno-Tempini ML, Warren JD, Rohrer JD. Speech and language therapy approaches to managing primary progressive aphasia. Pract Neurol 2020;20:154-161.
