# Supplementary material for: Long-Term Therapy With Transcranial Magnetic Stimulation in Primary Progressive Aphasia: A Randomized Clinical Trial
Source: JAMA Netw Open. 2025 Aug 11;8(8):e2526129. doi: 10.1001/jamanetworkopen.2025.26129 (PMC12340657; doi:10.1001/jamanetworkopen.2025.26129)
Supplement: Supplement 2. — eTable 1. Summary of Naming Hierarchy for PPA eTable 2. Baseline Demographic and Clinical Characteristics (Per Protocol Analysis for the Primary Outcome) eTable 3. Mean (SE) of Cognitive and Language Assessment at Baseline eTable 4. Comparison Between Participants Who Completed and Did Not Complete the Treatment eTable 5. Comparison of Participants Who Underwent Complete Versus Incomplete Outcome Assessments eTable 6. Summary of Treatment Outcomes at 3 and 6 Months eTable 7. Summary of IDDD Language Outcomes at 3 and 6 Months eTable 8. Raw Scores for Primary and Secondary Outcomes at Baseline, 3 Months, and 6 Months eTable 9. Adverse Events eFigure. Individual Changes in the Secondary Outcomes eAppendix. Study Visit Procedures eReferences [file jamanetwopen-e2526129-s002.pdf]

Supplemental Online Content

Fernández-Romero L, Cabrera-Martin MN, Delgado-Alonso C, et al. Long-term therapy with transcranial magnetic stimulation in primary progressive aphasia: a randomized clinical trial. *JAMA Netw Open*. 2025;8(8):e2526129. doi:10.1001/jamanetworkopen.2025.26129

eTable 1. Summary of Naming Hierarchy for PPA
eTable 2. Baseline Demographic and Clinical Characteristics (Per Protocol Analysis for the Primary Outcome)
eTable 3. Mean (SE) of Cognitive and Language Assessment at Baseline
eTable 4. Comparison Between Participants Who Completed and Did Not Complete the Treatment
eTable 5. Comparison of Participants Who Underwent Complete Versus Incomplete Outcome Assessments
eTable 6. Summary of Treatment Outcomes at 3 and 6 Months
eTable 7. Summary of IDDD Language Outcomes at 3 and 6 Months
eTable 8. Raw Scores for Primary and Secondary Outcomes at Baseline, 3 Months, and 6 Months
eTable 9. Adverse Events
eFigure. Individual Changes in the Secondary Outcomes
eAppendix. Study Visit Procedures
eReferences.

This supplemental material has been provided by the authors to give readers additional information about their work.

© 2025 Fernández-Romero L et al. *JAMA Network Open*.

eTable 1. Summary of Treatment Steps Based on Lexical Retrieval Treatment (Language Intervention) Utilized in the Current Study[1, 2, 3]

Semantic Feature Analysis	• Prompt semantic description of object with "Tell me about it."<br>• Clinician then prompts with follow-up questions to elicit important semantic features associated with the object.<br>• Clinician provides summary of participant's description<br>• Participant asked to recall name of object.
Orthographic /Phonemic Self-Cue	• Clinician prompts written production of the target word, "Can you write the word?"<br>• If unable to do so, clinician prompts for initial phoneme, "Can you say the first sound?"
Written and spoken repetition	• Clinician prompts participant to copy and read written word three times
Semantic plausibility judgments	• Clinician asks participant yes/no questions regarding semantic features of target (x5)
Recall	• Clinician asks participant to recall two important semantic features and to write and say the name from memory once
Description of a complex picture	• Clinician provides a complex image with the target included in the scene and asks participant to describe the picture using the target word

© 2025 Fernández-Romero L et al. *JAMA Network Open*.

eTable 2. Baseline Demographic and Clinical Characteristics (Per Protocol Analysis for the Primary Outcome)

		Active group (n=40)	Sham group (n=18)	U (p-value)
Demographic				
Age, mean (SD), years		71.52 (8.33)	71.33 (9.02)	346 (0.814)
Sex, Females		30 (75.0%)	8 (44.4%)	5.13 (0.024)
Race, White		40 (100%)	18 (100%)	-
Education, years		13.55 (5.10)	13.72 (4.04)	358 (0.979)
Clinical				
Time since symptom onset, months		29.33 (21.97)	27.87 (17.305)	3.121 (0.786)
PPA variant	nfvPPA*	16 (40.0%)	6 (33.3%)	0.306 (0.858)
	svPPA	7 (17.5%)	3 (16.7%)	
	lvPPA	17 (65.4%)	9 (50.0%)	
ACE-III (total score)		55.92 (18.85)	42.28 (21.63)	258 (0.086)
MLSE		79.13 (11.39)	75.39 (14.82)	318 (0.485)
NPI		9.03 (12.01)	8.39 (13.34)	308 (0.384)
IDDD		47.32 (13.34)	45.50 (11.05)	358 (0.980)

*Six patients had some degree of motor speech impairment (apraxia of speech/dysarthria): 3 in the active group, and 3 in the sham group.

Abbreviations: ACE: Addenbrooke's Cognitive Examination; IDDD: Interview for Deterioration of Daily Living in Dementia; MLSE: Mini-Linguistic State Examination; NPI: Neuropsychiatric Inventory; PPA: primary progressive aphasia.

© 2025 Fernández-Romero L et al. *JAMA Network Open*.

eTable 3. Mean (SE) of Cognitive and Language Assessment at Baseline

	Active group (N=42)	Sham group (N=21)	U (p-value)
	Cognitive assessment		
Span Forward	4.60 (1.380)	3.95 (2.037)	368.5 (0.883)
Span Backward	2.64 (1.32)	1.90 (1.33)	468.5 (0.057)
Corsi Forward	4.33 (1.50)	4.10 (1.57)	406.00 (0.428)
Corsi Backward	3.19 (1.56)	2,33 (1.90)	459.00 (0.088)
TMT A (sec.)	120.36 (69.89)	194.71 (243.63)	326.00 (0.568)
TMT B (sec.)	565.45 (351.38)	632.14 (359.84)	338.00 (0.693)
SDMT	18.07 (12.158)	12.43 (12.21)	463.00 (0.083)
ROCF Copy (/36)	24.39 (10.03)	19.92 (12.93)	435.00 (0.207)
ROCF Copy Time (sec.)	233.38 (126.92)	384.86 (260.99)	238.00 (0.040)
ROCF 3min Recall (/36)	9.64 (6.85)	4.95 (4.92)	503.5 (0.016)
ROCF 30min Recall (/36)	8.36 (7.14)	4.50 (5.52)	479.00 (0.044)
ROCF Recognition	16.60 (5.43)	13.43 (8.21)	404.5 (0.450)
Stroop Color	46.00 (20.18)	42.75 (23.86)	390.5 (0.608)
Stroop Word	25.55 (14.43)	16.30 (15.32)	471.00 (0.062)
Stroop Interference	11.52 (11.67)	6.45 (11.26)	477.00 (0.045)
ToL	1.52 (2.12)	0.67 (1.46)	445.00 (0.105)
VOSP Object decision (/20)	15.14 (3.26)	13.76 (5.03)	398.50 (0.515)
VOSP Progressive silhouettes	13.29 (4.06)	10.24 (6.07)	480.00 (0.043)
VOSP Position discrimination (/20)	18.62 (1.84)	17.76 (4.48)	361.00 (0.979)
VOSP Number location (/10)	7.45 (2.95)	6.05 (4.01)	394.50 (0.555)
Fluency (animals)	6.38 (4.87)	4.57 (2.82)	411.00 (0.388)
Fluency (P)	6.48 (3.58)	4.05 (3.77)	500.50 (0.018)
JLO (/30)	16.12 (9.28)	12.86 (11.71)	392.50 (0.582)
	Language assessment		
Reading Words (/24)	21.48 (4.92)	18.24 (8.44)	421.00 (0.278)
Reading Capitalized words (/24)	18.93 (5.27)	15.81 (8.17)	410.50 (0.393)
Reading Foreign words (/24)	12.52 (8.32)	8.76 (8.28)	434.00 (0.212)
Reading non-words(/24)	18.52 (5.96)	14.90 (9.11)	440.50 (0.173)
Initial phoneme omisión (/12)	6.43 (4.41)	4.81 (4.33)	433.50 (0.212)
Spelling words (/12)	3.69 (3.68)	2.57 (3.73)	432.50 (0.211)

© 2025 Fernández-Romero L et al. *JAMA Network Open*.

Repetition non-words (/12)	6.02 (3.50)	5.29 (4.03)	369.00 (0.879)
Semantic Association (/20)	18.67 (1.90)	17.14 (5.88)	386.50 (0.631)
Picture Naming (/20)	9.69 (6.53)	7.10 (6.17)	446.50 (0.145)
Word-picture matching (/20)	19.40 (1,28)	18.57 (3.14)	393.00 (0.480)
Synonyms (/20)	15.71 (5.28)	12.33 (7.002)	468.50 (0.066)
Actions Naming (/20)	14.95 (4.19)	11.57 (6.77)	431.00 (0.227)
Picture-action matching (/20)	18.55 (2.28)	16.71 (4.81)	440.00 (0.147)
Sentence comprehension (/20)	12.36 (5.83)	11.10 (6.27)	399.50 (0.506)
Verb tense concordance (/80)	45.86 (32.50)	20.43 (30.64)	503.50 (0.013)
Fluency (actions)	7.55 (4.23)	5.10 (5.56)	473.50 (0.056)
Syllables repetition (/8)	7.02 (1.22)	6.45 (2.41)	344.00 (0.773)
Two syllables repetition (/8)	6.38 (2.25)	5.55 (2.83)	410.50 (0.374)
Non-words repetition (/8)	5.26 (2.82)	4.85 (2.85)	369.00 (0.877)
2 two-syllables words repetition (/8)	5.38 (2.40)	4.75 (2.67)	391.00 (0.598)
Words repetition (/10)	9.19 (1.45)	9.35 (1.04)	326.00 (0.492)
Sentence repetition (/60)	46.64 (14.92)	32.65 (22.82)	451.00 (0.125)

Abbreviations: TMT: Trail Making Test, SDMT: Symbol Digit Modalities Test; ROCF: Rey-Osterrieth Complex Figure; ToL: Tower of London; VOSP: Visual Object and Space Perception Battery; JLO: Judgment of Line Orientation.

© 2025 Fernández-Romero L et al. *JAMA Network Open*.

eTable 4. Comparison Between Participants Who Completed and Did Not Complete the Treatment

Variable	Group that completed (n=60)	Group that did not complete (n=3)	Mann-Whitney U / Test	p-value (2-tailed)
Sex, female (%)	59 (98.3)	2 (66.7)		1.000
Age (SD)	71.50 (8.47)	77.33 (2.08)	46.500	0.160
Years of education (SD)	13.75 (4.75)	12.67 (4.62)	87.500	0.771
Baseline ACE-III Total (SD)	51.97 (19.85)	28.67 (24.17)	37.000	0.087
Active/sham group	41/19	1/2		0.256

Abbreviations: SD: standard deviation; ACE: Addenbrooke's Cognitive Examination.

eTable 5. Comparison of Participants Who Underwent Complete Versus Incomplete Outcome Assessments

Variable	All outcomes available (n=58)	Missing outcomes (n=5)	Mann-Whitney U / Test	p-value (2-tailed)
Sex, female (%)	38 (65.52)	1 (20.0)		0.657
Age (SD)	71.50 (8.47)	77.33 (2.08)	109.000	0.359
Years of education (SD)	13.75 (4.75)	12.67 (4.62)	120.500	0.509
Baseline ACE-III Total (SD)	51.97 (19.85)	28.67 (24.17)	78.000	0.088
Active/sham group	40/18	2/3		0.323

Abbreviations: SD: standard deviation; ACE: Addenbrooke's Cognitive Examination.

© 2025 Fernández-Romero L et al. *JAMA Network Open*.

eTable 6. Summary of Treatment Outcomes at 3 and 6 Months

Outcome	Adjusted Mean		F	p	η²
	a-TMS	s-TMS			
Primary outcome					
SUVR (6 months)	0.784	0.766	4.17	0.046	0.077
Secondary outcomes					
MLSE (6 months)	79.06	71.35	11.07	0.002	0.173
MLSE (3 months)	77.54	69.92	6.612	0.013	0.111
Naming (6 months)	143.81	119.99	15.97	<0.001	0.238
Naming (3 months)	136.57	121.77	8.59	0.005	0.135
WPM (6 months)	62.73	59.32	0.315	0.578	0.008
WPM (3 months)	65.46	59.57	1.57	0.217	0.036
IDDD (6 months)	43.54	48.94	4.10	0.048	0.073
IDDD (3 months)	45.81	47.62	0.701	0.406	0.013
NPI (6 months)	6.99	11.25	4.30	0.043	0.074
NPI (3 months)	7.38	11.39	3.51	0.066	0.059

Abbreviations: a-TMS: active transcranial magnetic stimulation; s-TMS: sham transcranial magnetic stimulation; MLSE: Mini-Linguistic State Examination; IDDD: Interview for Deterioration of Daily Living in Dementia; WPM: Words per Minute; NPI: Neuropsychiatric Inventory.

© 2025 Fernández-Romero L et al. *JAMA Network Open*.

eTable 7. Summary of IDDD Language Items at 3 and 6 Months

	Adjusted Mean (a-TMS)	(s-TMS)	F	p	η²
IDDD Language Total (6 months)	11.05	12.05	0.286	0.595	0.005
IDDD Language Total (3 months)	11.23	12.97	3.313	0.074	0.055
Item 24: Reading (6 months)	1.52	2.17	15.546	<0.001	0.227
Item 24: Reading (3 months)	1.62	2.17	9.397	0.003	0.146
Item 25: Writing (6 months)	2.00	2.61	6.806	0.012	0.114
Item 25: Writing (3 months)	2.11	2.27	0.668	0.417	0.012
Item 26: Conversation Initiation (6 months)	1.86	1.97	1.517	0.223	0.028
Item 26: Conversation Initiation (3 months)	1.92	2.21	1.947	0.168	0.034
Item 27: Verbal Expression (6 months)	2.17	2.44	0.180	0.673	0.003
Item 27: Verbal Expression (3 months)	2.21	2.38	1.504	0.225	0.027
Item 28: Conversational Attention (6 months)	1.76	1.89	3.753	0.058	0.066
Item 28: Conversational Attention (3 months)	1.91	2.14	1.017	0.318	0.018
Item 29: Comprehension of Spoken Language (6 months)	1.73	2.22	10.910	0.002	0.171
Item 29: Comprehension of Spoken Language (3 months)	1.82	2.28	6.884	0.011	0.111

Abbreviations: a-TMS: active transcranial magnetic stimulation; s-TMS: sham transcranial magnetic stimulation. **Language-related items:** 24. Do you have to help him/her with reading? 25. Do you have to help him/her write a letter, a postcard or fill in a form? 26. Is he/she able to initiate a conversation with another person as frequently as before? 27. Do

© 2025 Fernández-Romero L et al. *JAMA Network Open*.

you have to help him/her to express him/herself verbally? 28. Is he/she able to maintain attention in a conversation with another person as frequently as before? 29. Do you have to help him/her understand spoken language?

© 2025 Fernández-Romero L et al. *JAMA Network Open*.

eTable 8. Raw Scores for Primary and Secondary Outcomes at Baseline, 3 Months, and 6 Months

Outcome	A-TMS (Baseline)	S-TMS (Baseline)	A-TMS (3months)	S-TMS (3months)	A-TMS (6months)	S-TMS (6months)
SUVR	0.827±0.005	0.730±0.009	-	-	0.812±0.012	0.702±0.023
MLSE	78.92±1.72	73.42±3.55	79.11±1.94	65.10±4.45	79.42±1.96	67.55±4.23
Naming	149.47±11.41	108.23±18.02	149.80±11.87	92.55±18.17	152.45±12.08	98.11±18.54
WPM	70.41±5.33	56.31±8.76	62.94±4.70	49.45±6.72	63.51±4.84	46.72±8.14
IDDD	48.02±2.21	44.95±2.27	46.21±1.94	47.90±2.45	44.78±1.91	48.61±2.43
NPI	8.92±1.82	7.76±2.71	7.54±1.43	10.45±3.24	7.00±1.43	10.61±2.78

Abbreviations: SUVR = Standardized Uptake Value Ratio (FDG-PET); MLSE = Mini Linguistic State Examination; WPM = Words Per Minute; IDDD = Interview for Deterioration in Daily Living Activities in Dementia; NPI = Neuropsychiatric Inventory.

© 2025 Fernández-Romero L et al. *JAMA Network Open*.

eTable 9. Adverse Events

Adverse event	Treatment, No. (%) of Participants	
	a-TMS (N=42)	s-TMS (N=21)
	Probably related	
Treatment site discomfort	1 (2.38)	0
	Unrelated	
Cancer	0	2 (9.52)
Hypercholesterolemia	0	1 (4.76)
Asthma	0	1 (4.76)
Urinary Tract Infection	1 (2.38)	0
Neuropathic Pain	1 (2.38)	0
Constipation	1 (2.38)	0
Insomnia	1 (2.38)	0
Gastroenteritis	1 (2.38)	0
Depression	0	1 (4.76)
Herpes Zoster	1 (2.38)	0

Abbreviations: a-TMS: active Transcranial Magnetic Stimulation; s-TMS: sham Transcranial Magnetic Stimulation.

© 2025 Fernández-Romero L et al. *JAMA Network Open*.

eFigure. Individual Changes in the Secondary Outcomes

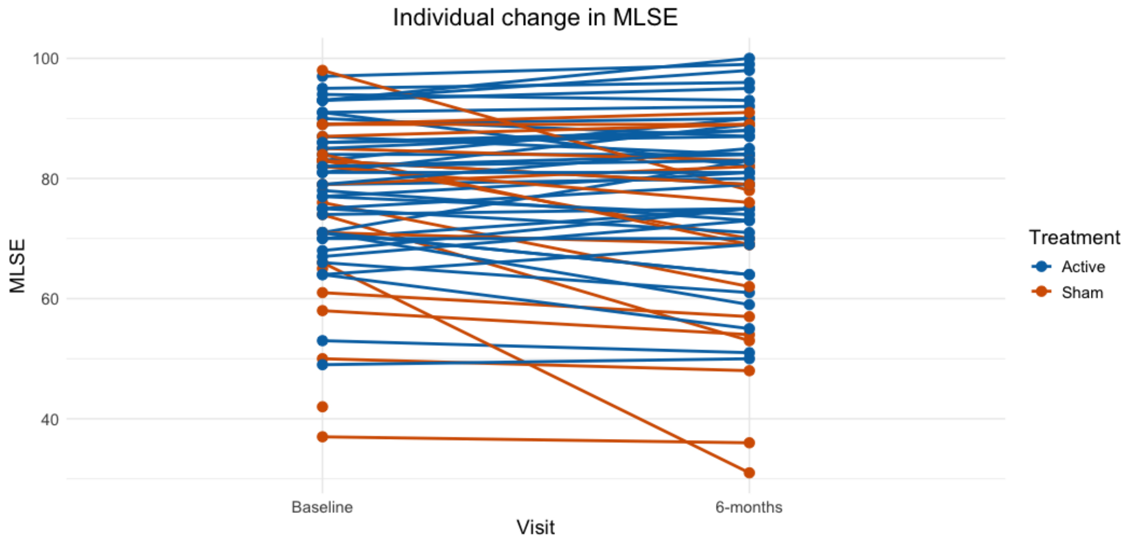

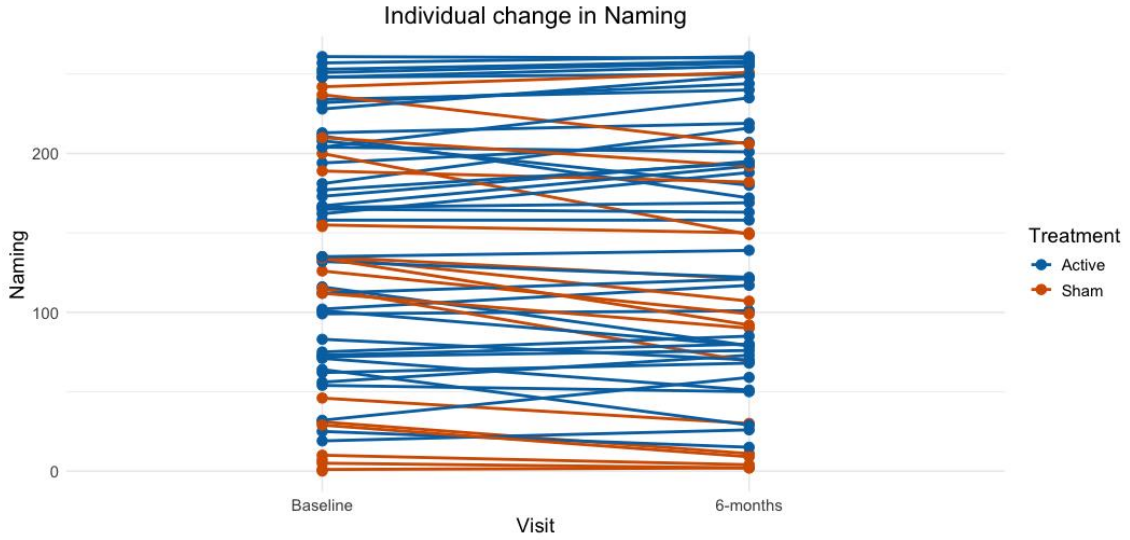

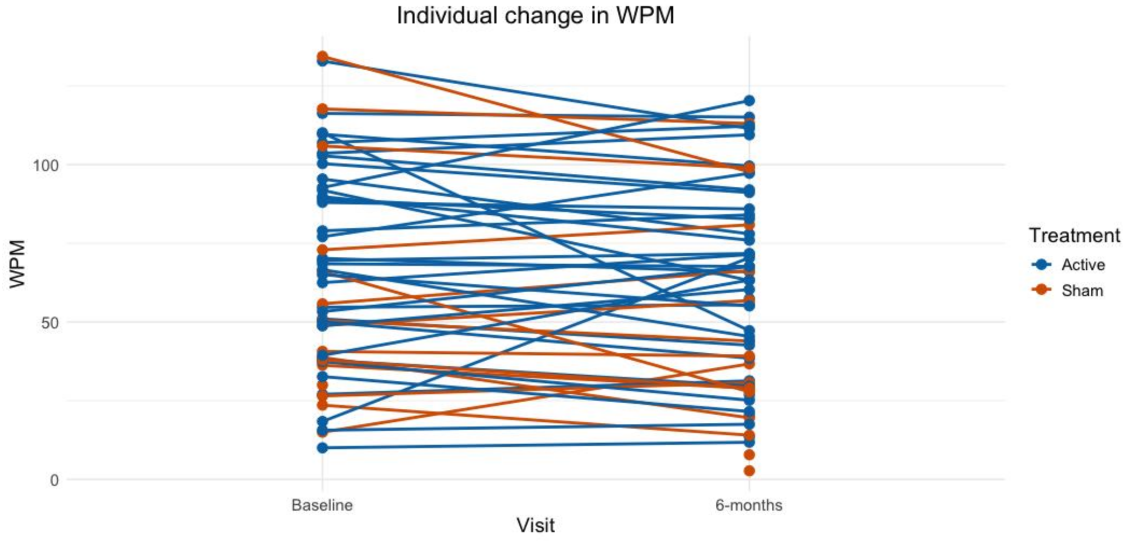

© 2025 Fernández-Romero L et al. *JAMA Network Open*.

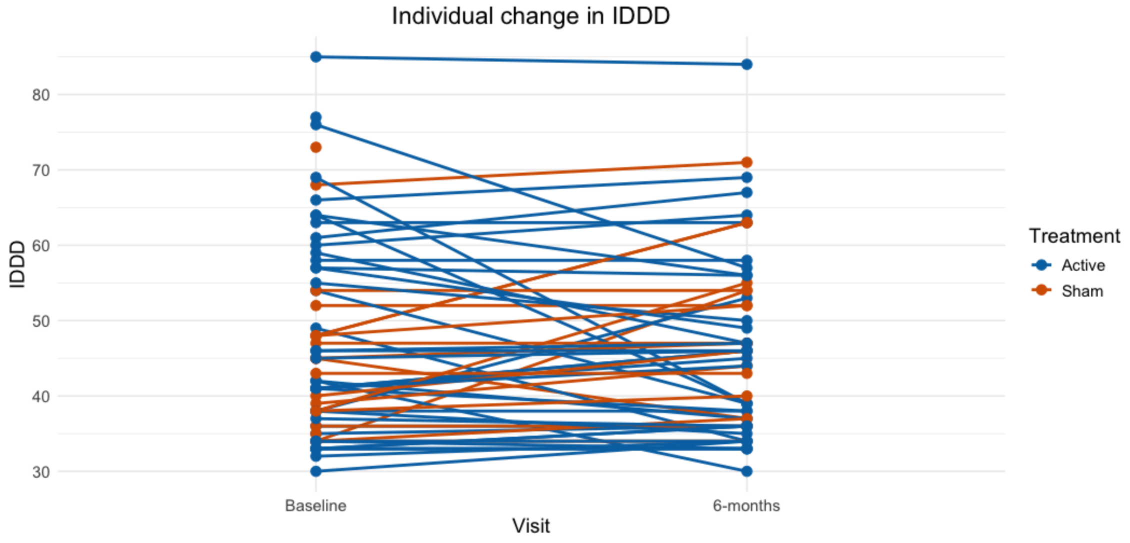

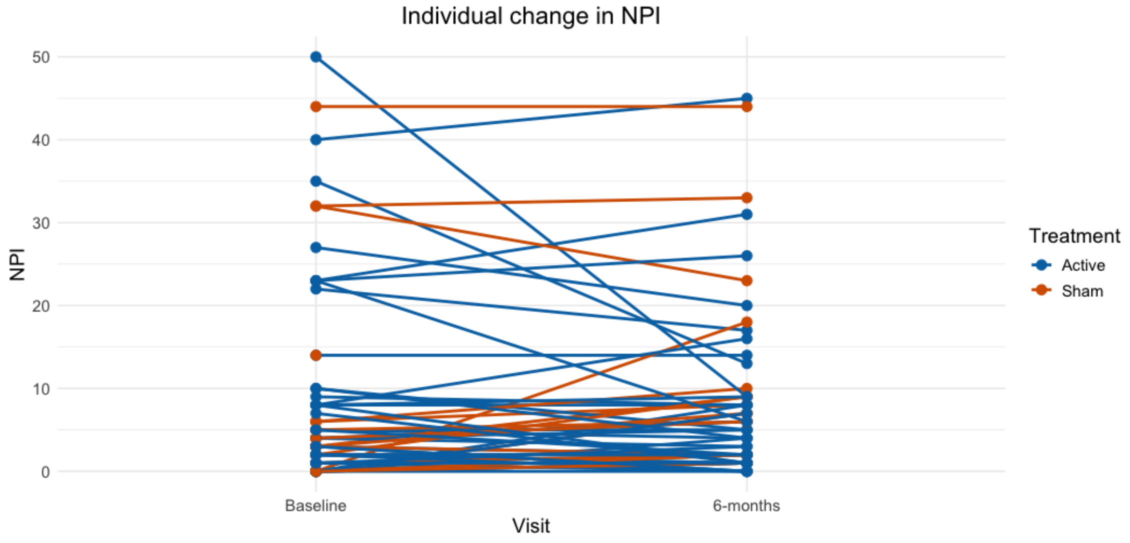

Line plot representing the individual secondary outcomes before (Baseline) and at the end of the trial (6-months) for the active TMS group (blue) and for the sham TMS group (orange). MLSE: Mini Linguistic State Examination; WPM: Word Per Minute; NPI: Neuropsychiatric Inventory; IDDD: Interview for Deterioration of Daily Living in Dementia; TMS: Transcranial Magnetic Stimulation.

© 2025 Fernández-Romero L et al. *JAMA Network Open*.

eAppendix. Study Visit Procedures

The initial screening visit occurred at the Department of Neurology of the Hospital Clínico San Carlos, Madrid, Spain. Clinical information was collected from the patient and caregivers, including date of birth, sex, years of formal education, year of PPA diagnosis, relevant prior medical conditions and medication. Patients who met the eligibility criteria were informed about the study verbally and in writing. Once the patient decided to participate in the study, they signed two copies of the informed consent. Within 30 days of this visit, treatment visits began with a baseline visit that included neurologic, language, and cognitive assessments, and FDG-PET. At this time, the patient was randomized into one of the two study arms. Before beginning TMS treatment, a pre-treatment assessment was conducted and then repeated at 3 and 6 months, the end of the treatment, including: MLSE, naming word list, number of words per minute in spontaneous speech, NPI, and IDDD. In the final visit, FDG-PET was also included.

1. **Screening visit**

Review inclusion and exclusion criteria

Clinical information: date of birth, sex, years of formal education, year of PPA diagnosis, relevant prior medical conditions, medication and family history

Clinical Dementia Rating (CDR) Scale score of 0-1

Informed consent

2. **Visit 1 (Baseline)**

Language and cognitive assessment

Concomitant medication assessment

Adverse event (AE) assessment (Interview and review study diary)

MLSE

Naming of a list of trained words

Words per minute in a spontaneous speech task

Functional activity assessed using the Interview for Deterioration in Daily Living Activities in Dementia (IDDD) scale

Neuropsychiatric symptoms assessed using the Neuropsychiatric Inventory (NPI).

FDG-PET

Brain MRI

© 2025 Fernández-Romero L et al. *JAMA Network Open*.

3. **Visit 2 (3 months from visit 1)**

Concomitant medication assessment

Adverse event (AE) assessment (Interview and review study diary)

MLSE

Naming of a list of trained words

Words per minute in a spontaneous speech task

Functional activity assessed using the Interview for Deterioration in Daily Living Activities in Dementia (IDDD) scale

Neuropsychiatric symptoms assessed using the Neuropsychiatric Inventory (NPI

4. **Visit 3 (6 months from visit 1)**

Concomitant medication assessment

Adverse event (AE) assessment (Interview and review study diary)

MLSE

Naming of a list of trained words

Words per minute in a spontaneous speech task

Functional activity assessed using the Interview for Deterioration in Daily Living Activities in Dementia (IDDD) scale

Neuropsychiatric symptoms assessed using the Neuropsychiatric Inventory (NPI).

FDG-PET

ASSESSMENTS

Visit 1 (Baseline)

1. Language assessment

Different tasks that assess language domains: Cookie Theft picture; Paradis narrative sequence picture; reading narrative text, words, capitalized words, foreign words, and non-words; omission of initial phoneme; spelling words; repetition of words, non-words, syllables and sentences; semantic association; naming pictures and actions; word-picture matching; synonyms; picture-action matching; orophonatory praxis; verb tense agreement; sentence comprehension.

© 2025 Fernández-Romero L et al. *JAMA Network Open*.

2. Cognitive assessment

Test included in NEURONORMA protocol:[4] memory span (forward and backward digit spans), the Corsi block-tapping test, Trail Making Test (TMT), Symbol Digit Modalities Test (SDMT), Visual Object and Space Perception Battery (VOSP; subtests for object decision, progressive silhouettes, position discrimination, and number location), Judgement of Line Orientation (JLO), Tower of London-Drexel University, Rey–Osterrieth Complex Figure Test (copy and memory), and Stroop Color–Word Interference Test.

3. Addenbrooke's cognitive examination III (ACE-III)

ACE-III is a general cognitive screening tool that assesses attention (temporal spatial orientation, 3-item registration, serial subtractions) memory (recall of 3 items, anterograde memory of a name and address, retrograde memory, and recall and recognition of a name and address) verbal fluency (words beginning with “p” and animals), language (comprehension of a complex command, sentence writing, single word repetition, proverb repetition, a 10-item confrontation naming task, a semantic task, and reading) and visuospatial skills (copy of intersecting infinity loops, copy of a cube, drawing of a clock, counting dots, and identifying incomplete letters).

Visit 1, 2 and 3 (week 0, week 12 and week 24)

1. Mini-Linguistic state examination (MLSE)

The MLSE is a brief screening tool designed to assess language impairments in various neurodegenerative disorders, particularly in PPA. It includes a series of tasks that evaluate different language processes: naming, syllable repetition, repeat and point, non-word repetition, semantic association, sentence comprehension, reading, sentence repetition, writing, and picture description. These tasks are grouped in five language domains: motor, phonology, semantics, syntax, and working memory, and scoring is done by classifying the errors made by the subject. The time of administration is approximately 20 min. The Spanish version of the MLSE has demonstrated good internal consistency (Cronbach’s α = 0.758) and excellent discriminant validity, with an area under the curve (AUC) of 0.99 for distinguishing patients with PPA from healthy controls.[5]

2. Naming of trained words

A list of 261 words from 8 different semantic categories: animals, food, objects, places, furniture, clothing, transportation, and body parts. Two consecutive sessions of oral naming of the list were conducted.

3. Words per minute in spontaneous speech

© 2025 Fernández-Romero L et al. *JAMA Network Open*.

The participant was shown a wordless book (Frog stories) and was asked to describe the story in detail for 3 minutes. The number of words per minute was calculated.

4. Interview for Deterioration in Daily Life in Dementia (IDDD)

IDDD is a scale designed to assess functional decline in daily and instrumental activities. It is a caregiver-administered tool. The Spanish version has demonstrated excellent internal consistency (Cronbach's α = 0.985) and strong test-retest reliability (intraclass correlation coefficient [ICC] = 0.94).[6]

5. Neuropsychiatric Inventory (NPI)

It is a caregiver-administered tool designed to assess neuropsychiatric symptoms. The NPI assesses 12 different symptom domains, which include: delusions, hallucinations, agitation/aggression, dysphoria/depression, anxiety, euphoria/elation, apathy/indifference, disinhibition, irritability/lability, aberrant motor behaviors, night-time behavioral disturbances and appetite/eating disturbances. The Spanish version of the NPI-Q has demonstrated test-retest correlations of r = 0.89 for the symptom severity scale and r = 0.90 for caregiver distress. It also shows strong convergent validity with the original NPI (r = 0.879 for symptoms; r = 0.92 for distress).[7]

Visit 1 and 3 (week 0 and week 24)

The participants underwent an FDG-PET and a blood test at the beginning and end of the study.

FDG-PET studies were performed using a Siemens Biograph TruePoint PET-CT scanner, which integrated a state-of-the-art detector with lutetium oxyorthosilicate crystals. Participants fasted for at least 6 hours prior to the scan. The dose was administered 30 minutes before image acquisition. Current European recommendations for the acquisition of FDG-PET brain studies were followed.[8]

FDG-PET preprocessing and analysis

FDG-PET images were pre-processed using the Statistical Parametric Mapping 12 program (Wellcome Trust Centre for Neuroimaging, Institute of Neurology, London). The images were manually realigned to the bi-commissural line. Normalization to the reference space of the Montreal Neurological Institute was conducted using a template specifically validated for FDG-PET imaging in dementia.[9]

© 2025 Fernández-Romero L et al. *JAMA Network Open*.

The analysis of cerebral metabolism was carried out using a broad region of interest that included much of the left hemisphere. This region of interest (see the Figure below, in red) was obtained by comparing, through voxel-based analysis, a cohort of 70 PPA participants evaluated at two time points, and it was the region of interest used for sample size calculation. The relative uptake in this area was calculated at baseline and after 6 months of the study, using the whole cerebellum as reference.

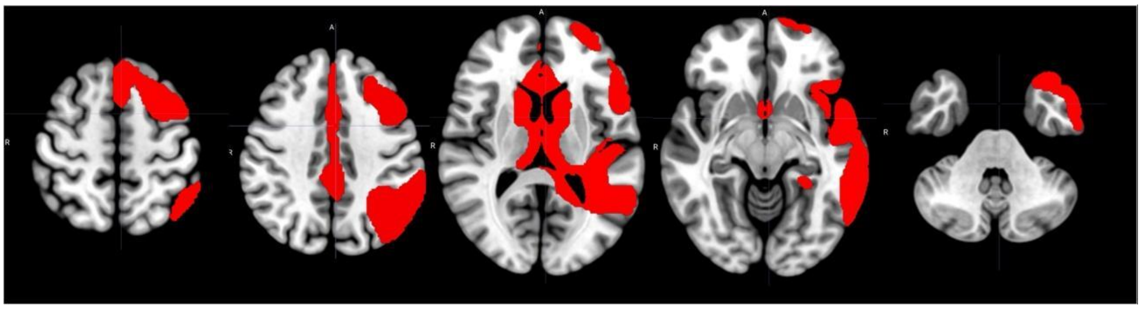

© 2025 Fernández-Romero L et al. *JAMA Network Open*.

eReferences

1. Henry, M. L., Rising, K., DeMarco, A. T., Miller, B. L., Gorno-Tempini, M. L., & Beeson, P. M. (2013). Examining the value of lexical retrieval treatment in primary progressive aphasia: Two positive cases. *Brain and Language*, *127*(2), 145–156. https://doi.org/10.1016/j.bandl.2013.05.018

2. Henry, M. L., Hubbard, H. I., Grasso, S. M., Dial, H. R., Beeson, P. M., Miller, B. L., & Gorno-Tempini, M. L. (2019). Treatment for word retrieval in semantic and logopenic variants of primary progressive aphasia: Immediate and long-term outcomes. *Journal of Speech, Language, and Hearing Research*, *62*(8), 2723–2749. https://doi.org/10.1044/2018_JSLHR-L-18-0144

3. Grasso, S. M., Peña, E. D., Kazemi, N., Mirzapour, H., Neupane, R., Bonakdarpour, B., Gorno-Tempini, M. L., & Henry, M. L. (2021). Treatment for Anomia in Bilingual Speakers with Progressive Aphasia. *Brain Sciences*, *11*(11), 1371. https://doi.org/10.3390/brainsci11111371

4. Peña-Casanova J, Blesa R, Aguilar M, et al. Spanish multicenter normative studies (NEURONORMA project): Methods and sample characteristics. *Arch Clin Neuropsychol.* 2009;24(4):307–319.

5. Matias-Guiu, J. A., Pytel, V., Hernández-Lorenzo, L., Patel, N., Peterson, K. A., Matías-Guiu, J., ... & Cuetos, F. (2021). Spanish version of the mini-linguistic state examination for the diagnosis of primary progressive aphasia. *Journal of Alzheimer's Disease*, *83*(2), 771–778.

6. Böhm, P., Peña-Casanova, J., Aguilar, M., Hernández, G., Sol, J. M., Blesa, R., & NORMACODEM Group. (1998). Clinical validity and utility of the interview for deterioration of daily living in dementia for Spanish-speaking communities. *International Psychogeriatrics*, *10*(3), 261–270.

7. Boada, M., Cejudo, J. C., Tarraga, L., López, O. L., & Kaufer, D. (2002). Neuropsychiatric inventory questionnaire (NPI-Q): Spanish validation of an abridged form of the Neuropsychiatric Inventory (NPI). Neurología (Barcelona, Spain), 17(6), 317–323.

8. Varrone A, Asenbaum S, Vander Borght T, et al. EANM procedure guidelines for PET brain imaging using [18F] FDG, version 2. Eur J Nucl Med Mol Imaging. 2009;36:2103–2110.

9. Della Rosa, P. A., Cerami, C., Gallivanone, F., Prestia, A., Caroli, A., Castiglioni, I., et al. (2014). A standardized [18F]-FDG-PET template for spatial normalization in statistical parametric mapping of dementia. Neuroinformatics, 12, 575–593. https://doi.org/10.1007/s12021-014-9235-4

© 2025 Fernández-Romero L et al. *JAMA Network Open*.
