# Supplementary material for: Long-Term Therapy With Transcranial Magnetic Stimulation in Primary Progressive Aphasia: A Randomized Clinical Trial
Source: JAMA Netw Open. 2025 Aug 11;8(8):e2526129. doi: 10.1001/jamanetworkopen.2025.26129 (PMC12340657; doi:10.1001/jamanetworkopen.2025.26129)
Supplement: Supplement 3. — Data Sharing Statement [file jamanetwopen-e2526129-s003.pdf]

Data Sharing Statement

Fernández-Romero. Long-Term Therapy With Transcranial Magnetic Stimulation in Primary Progressive Aphasia. *JAMA Netw Open*. Published August 11, 2025.
doi:10.1001/jamanetworkopen.2025.26129

Data
Additional Information: RECONNECT; https://clinicaltrials.gov/study/NCT05842473?term=NCT05842473&rank=1; ClinicalTrials.gov identifier: NCT05842473
Data available: Yes
Data types: Deidentified participant data
How to access data: jordi.matias-guiu@salud.madrid.org
When available: With publication

Supporting Documents
Document types: None

Additional Information
Who can access the data: researchers whose proposed use of the data has been approved
Types of analyses: for any purpose
Mechanisms of data availability: with investigator support after approval of a proposal.
